# Multidimensional design of carbon-supported Ru-based catalysts: a journey to hydrogen evolution reaction performance breakthroughs

**DOI:** 10.1039/d5sc06468f

**Published:** 2025-11-18

**Authors:** Zonglin Liu, Shan Zhao, Bingchen Liu, Pengfei Wang, Tingfeng Yi

**Affiliations:** a School of Materials Science and Engineering, Northeastern University Shenyang 110819 PR China tfyihit@163.com; b Key Laboratory of Dielectric and Electrolyte Functional Material Hebei Province, School of Resources and Materials, Northeastern University at Qinhuangdao Qinhuangdao 066004 PR China

## Abstract

The urgent need for global carbon neutrality positions green hydrogen, produced *via* water electrolysis with zero emissions, as a crucial component of the clean energy framework. Ruthenium (Ru)-based catalysts exhibit intrinsic activity similar to that of platinum (Pt)-based catalysts while offering considerable cost benefits, positioning them as optimal alternatives to Pt-based catalysts for hydrogen evolution reactions (HER). However, their electrochemical stability and activity under varying pH conditions continue to present challenges. This review systematically analyzes the structure–activity relationships and multilevel design strategies of carbon-supported Ru-based catalysts in HER. The design principles related to reaction environments (acidic, alkaline, and neutral) are examined thoroughly, emphasizing the optimization of carbon supports and their synergistic effects with Ru active sites. The review provides a summary of active site modulation strategies, with a focus on optimizing carbon supports through structural design, defect engineering, heteroatom doping, and surface chemistry. It systematically outlines strategies for optimizing the electronic structure of supports, enhancing mass transfer, improving structural stability, and elucidating interfacial electron transfer and stabilization mechanisms. It examines the primary applications of computational tools, such as density functional theory (DFT), finite element simulation, and machine learning, in the design of catalyst electronic structures, optimization of electrodes, and prediction of performance. This review integrates theoretical mechanisms with practical design to establish a systematic framework encompassing environmental adaptation, active site engineering, support optimization, and intelligent design. The objective is to advance the development of high-performance, stable carbon-supported Ru-based catalysts for HER, offering researchers a robust theoretical framework and practical guidance.

## Abstract 2

This review comprehensively analyzes Ru-based catalyst design concepts and shows how supports optimize charge transfer channels, proton transport networks, and interfaces to meet the activity-stability trade-off.

## Graphical abstract

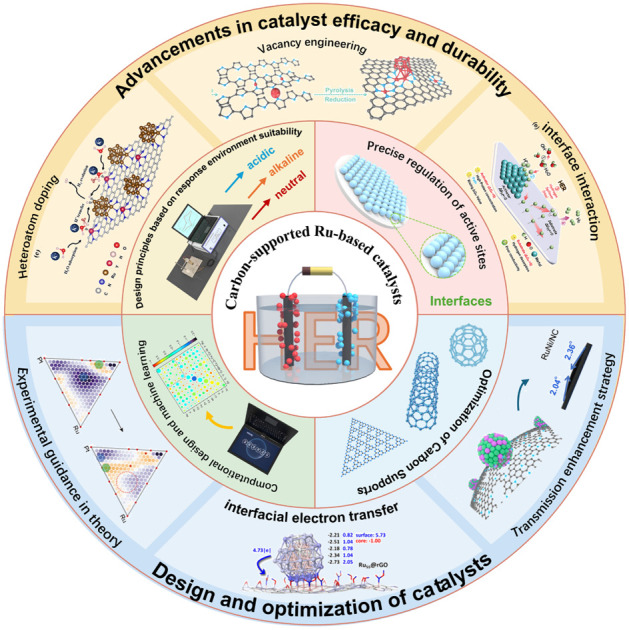

## 1 Introduction

In the context of the worldwide transition towards low-carbon energy systems, green hydrogen has become a crucial carbon-neutral energy support.^1–3^ The production of green hydrogen on a large scale has become essential for reaching carbon neutrality.^4–6^ Water electrolysis, characterized by its operational flexibility and environmental compatibility, is regarded as an optimal method for producing green hydrogen.^7,8^ The kinetics of the hydrogen evolution reaction (HER) in water electrolysis play a crucial role in influencing the overall energy conversion efficiency.^9–11^ Platinum (Pt)-based catalysts exhibit remarkable HER activity.^12^ However, their limited availability and high expense make them inappropriate for widespread use in industrial electrolyzers.^13,14^ Ruthenium (Ru) has gained recognition as a cost-effective alternative to Pt-group metals. With its near-thermoneutral hydrogen adsorption free energy and swift Volmer–Tafel kinetics, Ru exhibits intrinsic catalytic potential comparable to that of Pt.^15–17^ Nonetheless, Ru metals encounter considerable electrochemical obstacles, such as dissolution loss in acidic conditions, surface oxidative passivation in alkaline settings, and active site deactivation in neutral environments.^18–20^ The challenges presented underscore the fundamental struggle to achieve a balance between activity and stability in individual metal components. This critical limitation underscores the necessity of a synergistic approach in designing carbon supports and Ru to surpass performance thresholds. Highly conductive carbon supports, such as graphene, carbon nanotubes, and porous carbon, effectively prevent Ru particle agglomeration through physical domain-limiting effects and also influence the positioning of Ru's d-band center due to their distinctive sp^2^ electronic structure.^21,22^ This dual role enhances the adsorption characteristics of reactive intermediates and guarantees stable, long-term operation throughout the entire pH spectrum.

**Fig. 1:**
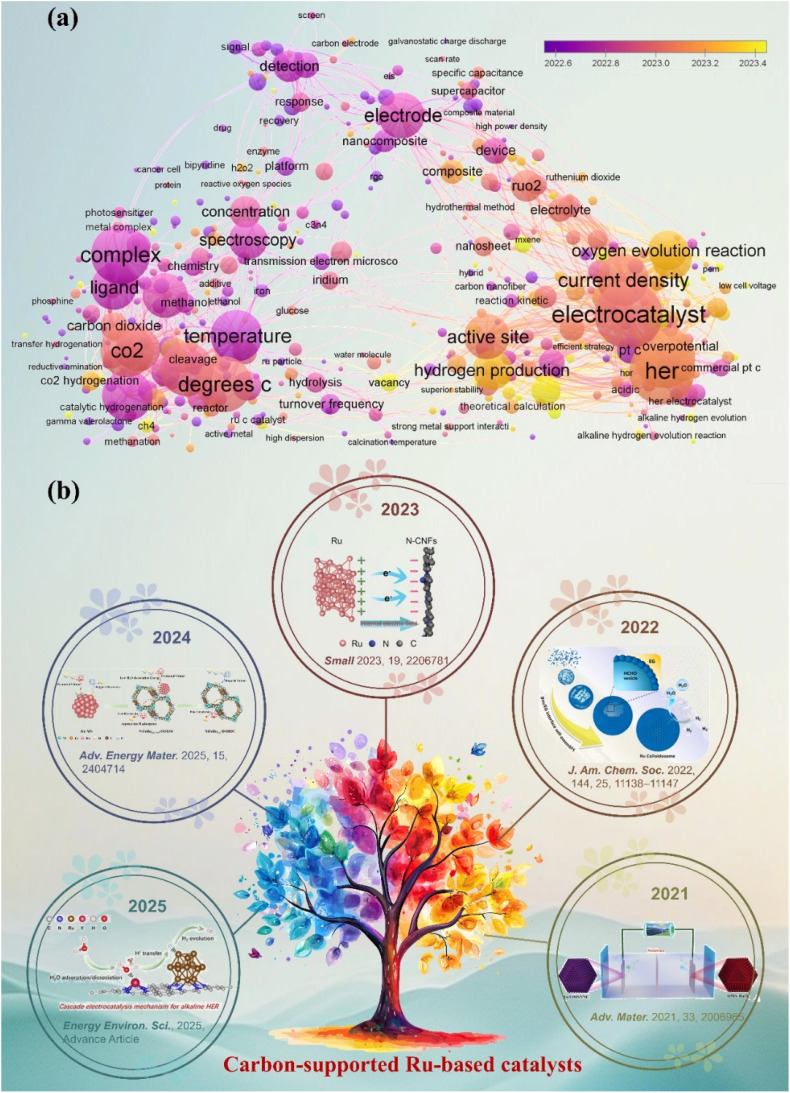
(a) Network map of scientific research priorities regarding carbon-supported Ru-based catalysts, derived from a bibliometric analysis of the Web of Science core collection (2020–2025) and visualized with VOS viewer. The size and color of the nodes indicate the frequency and thematic cluster of the keywords, respectively. The interconnecting lines and proximity signify the strength of co-occurrence. The yellow core node indicates the central role of the HER. (b) Timeline of outstanding representative works on carbon-supported Ru-based catalysts in the HER. Reproduced with permission from ref. 32. Copyright 2025, Royal Society of Chemistry. Reproduced with permission from ref. 33. Copyright 2025, Wiley-VCH. Reproduced with permission from ref. 34. Copyright 2023, Wiley-VCH. Reproduced with permission from ref. 35. Copyright 2022, American Chemical Society. Reproduced with permission from ref. 36. Copyright 2021, Wiley-VCH.

As illustrated in Fig. 1a, the network map of scientific research priorities offers a comprehensive overview of the current landscape in the HER field. The HER is recognized as a prominent area of inquiry, highlighted by a yellow core node. The investigation primarily focuses on essential performance factors, such as catalyst active site design, current density enhancement, and overpotential minimization.^23–25^ The reaction mechanism and the adaptability of catalysts to various electrolyte environments, such as alkaline HER and neutral medium systems, are increasingly becoming significant areas of focus.^26–28^ Notably, carbon materials show great promise due to their wide range of electrode applications.^29–31^ Over the past five years, there has been a significant surge in studies focused on carbon-supported Ru-based catalysts. Significant advancements have been made in the precise regulation of active sites at the atomic scale, the optimization of adaptability across multiple pH levels, and the integration at the device level (Fig. 1b).^32–36^ This trend is driving a profound integration of carbon catalyst synergies, including electron coupling and defect engineering, across advanced synthesis, atomic design, mechanistic analysis, and device applications. This pattern underscores the necessity for a comprehensive overview of the structure–activity relationships and design principles of carbon-supported Ru-based catalysts, along with their strategic importance. With the increasing focus on carbon-supported Ru-based catalysts, a systematic summary of their multi-dimensional design and intrinsic mechanisms is urgently required. Recent reviews have concentrated on enhancing the intrinsic activity of Ru or improving the performance of single pH systems. However, there is a notable lack of design guidelines for the synergistic interaction between carbon supports and Ru activity centers across acidic, basic, and neutral environments. Furthermore, there is an insufficient systematic investigation into the construction and modulation of multi-scale Ru structures (single atoms, clusters, nanoparticles), especially in relation to optimizing carbon supports (such as surface chemistry and structural design) and their interaction with Ru sites. Although machine learning-assisted catalyst design has a transformative impact, there is a significant gap in the systematic review and understanding of the potential of advanced methods in Ru-based HER systems.

This review systematically constructs the design framework for carbon-supported Ru-based HER catalysts. The design principles for various reaction environments (acidic, alkaline, and neutral) are thoroughly discussed, with a clear delineation of the key points and requirements for catalyst design in each context. Secondly, the regulation of active sites is reviewed in detail, including the dispersion of single atoms to optimize coordination and electronic structure, the enhancement of electronic coupling and mass transfer between clusters and single atoms, and the design of heterostructures to control interfacial charge and intermediate adsorption, all aimed at achieving a significant improvement in catalytic performance. This review specifically examines the enhancement of carbon supports and their collaborative impacts with active centers. A comprehensive analysis is conducted on the characteristics and performance enhancement mechanisms of various classes of carbon supports. The effects of the surface chemistry of these supports, such as defect engineering and heteroatom doping, on reaction pathways and stability are thoroughly discussed. Subsequently, the methods for optimizing the electronic configuration of supports, enhancing mass transfer, and strengthening the stability of structures are comprehensively outlined. Additionally, the mechanisms governing interfacial electron transfer between the supports and the Ru active sites, as well as the stabilization of the nanostructures, are analyzed in depth. Finally, this review evaluates advanced applications of computational design and machine learning, highlighting the essential role of density functional theory (DFT) in electronic structure design, the use of finite element simulation for optimizing electrode structures, and the potential of machine learning in catalyst prediction and design. It elucidates how these approaches create a quantitative relationship between activity descriptors and performance metrics to facilitate rational design. This review integrates core themes of environmental adaptation, active site engineering, support optimization, and intelligent design to establish a systematic theoretical framework and technical guidelines for developing high-performance carbon-supported Ru-based HER catalysts. The objective is to offer valuable insights into experimental design and theoretical references, facilitating the advancement of this field towards greater accuracy and efficiency, while also providing robust support for the realization of effective energy conversion and storage.

## 2 Design principles of carbon-supported Ru-based catalysts for HER

### 2.1 Design principles based on response environment suitability

The HER is significantly affected by the reaction medium environment, which may be acidic, alkaline, or neutral, influencing both the reaction mechanisms and kinetic characteristics.^37–39^ The primary distinctions among these environments stem from differences in the concentration gradients of active species, including H^+^, H_2_O, and OH^−^, present in the electrolyte. The varied characteristics of the interfacial reaction pathways significantly influence the differences in HER performance and behavior under these specific electrolytic conditions.^40–42^

In an acidic environment, the elevated proton concentration promotes a swift reaction rate.^43,44^ Nonetheless, the harsh conditions present in a highly acidic environment led to equipment corrosion, requiring meticulous attention to material compatibility and system longevity in acidic HER applications.^45^ In alkaline conditions, the dissociation of water emerges as the rate-limiting step of the reaction, influenced by the elevated concentration of OH^−^ and the diminished concentration of protons.^46^ As a result, the kinetics of the HER is 2–3 orders of magnitude slower compared to that in acidic media.^47,48^ Nonetheless, the alkaline system proves to be more appropriate for extensive hydrogen production due to the enhanced stability of electrode materials.^19,49^ On the other hand, neutral media provides a blend of environmental advantages, sustainability, and economic efficiency.^50^ Nonetheless, the kinetics of their reactions are significantly slower, which requires enhanced catalyst performance.^51,52^ Although design strategies for various pH environments have been thoroughly investigated, a significant challenge persists in creating “all-pH” catalysts capable of stable operation under these fluctuating conditions. Current research predominantly restricts performance evaluation to individual, pure electrolytes, which does not reflect the intricate electrolyte compositions found in practical applications, including ions in seawater and contaminants in industrial wastewater.

#### 2.1.1 Design principles for acidic conditions

**Fig. 2:**
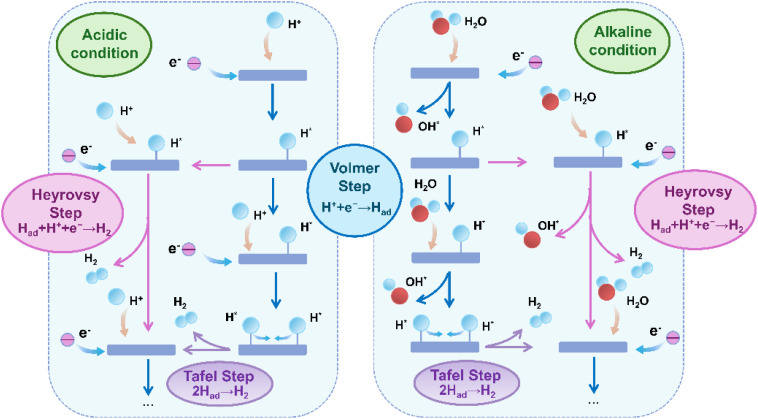
Schematic diagram of HER mechanism in different conditions. Under acidic conditions, the HER proceeds *via* the Volmer step (H^+^ + e^−^ → H_ad_), followed by either the Heyrovsky step (H_ad_ + H^+^ + e^−^ → H_2_) or the Tafel step (2H_ad_ → H_2_). In alkaline media, the process initiates with the water dissociation Volmer step (H_2_O + e^−^ → H_ad_ + OH^−^), which is then followed by the analogous Heyrovsky or Tafel recombination steps.

The acidic systems generally show enhanced intrinsic reaction kinetics, which can be linked to the increased proton concentration that lowers the activation energy barrier of the Volmer step (H^+^ + e^−^ → H_ad_).^43,53^ Nonetheless, the highly acidic conditions (pH < 1) are susceptible to the anodic dissolution of the Ru active center and the electrochemical corrosion of the carbon support, potentially leading to structural destabilization of the catalyst and a decline in performance.^54^ The HER mechanism under acidic conditions operates through a proton-coupled electron transfer pathway, primarily characterized by the Volmer step, which is succeeded by either the Heyrovsky step (H_ad_ + H^+^ + e^−^ → H_2_) or the Tafel step (2H_ad_ → H_2_) (Fig. 2).^55,56^ In conclusion, in a concentrated pure acidic electrolyte, the rate of the acidic HER is primarily influenced by H adsorption and H_2_ desorption kinetics, as the plentiful supply of H^+^ eliminates the necessity for the water dissociation step found in alkaline conditions. The main design objective for catalysts in these conditions is to optimize the hydrogen adsorption free energy (Δ*G*_H*_) and enhance electrical conductivity to achieve effective HER performance.^57,58^ The kinetics of H^+^ transport and the local solvation environment are critical factors influencing the overall reaction rate in non-ultrapure or highly concentrated industrial electrolytes. Optimizing the electronic structure is a vital approach for improving the performance of carbon-supported Ru-based catalysts in acidic conditions, with the goal of lowering the H* adsorption energy barrier. Reducing the size of Ru particles has been shown to enhance both the quantity and activity of active sites present on their surface. This process enhances the interactions between the metal and support, subsequently modifying the electron distribution and increasing the effectiveness for hydrogen adsorption. For example, Ma *et al.*^59^ developed an Ru-based catalyst (Ru/NPCS) that integrated both Ru nanoclusters and single atoms, demonstrating enhanced activity and mass activity in comparison to Pt catalysts in acidic environments. The improved performance was linked to the Ru nanoclusters facilitating hydrogen evolution through the Volmer–Tafel mechanism. Furthermore, the investigation revealed that there were no synergistic interactions present between the single-atom Ru species and the Ru clusters. This finding indicates that although Ru nanoclusters exhibit high activity, the single-atom Ru species do not serve as active sites for HER and instead assume a passive role in the reaction.

Moreover, improving stability and resistance to poisoning is critically important. Nitrogen-doped carbon-supported Ru catalysts exhibit remarkable stability and resilience against poisoning in acidic conditions. For example, Liu *et al.*^60^ developed Ru nanoparticle catalysts (Ru/NCDs) on nitrogen-doped carbon supports that exhibited exceptional catalytic activity and stability after thorough electrochemical testing in an acidic electrolyte. The observed performance can be largely ascribed to the integration of nitrogen atoms within the carbon matrix, which influenced the electronic structure and surface chemical environment of the support. The modifications probably improved the catalyst's ability to resist poisoning. At the same time, choosing carbon supports that possess a high specific surface area and an ideal pore structure can fully enhance the accessibility of active sites. For example, Long *et al.*^61^ effectively created a composite support (V_8_C_7_/C) for Ru nanoparticles through the derivatization of MOF (V-BDC). The Ru nanoparticles produced, averaging 2.3 nm in size, demonstrate a significant specific surface area. The porous carbon structure facilitates swift electron transfer pathways, while the V_8_C_7_ integrated within the carbon matrix electronically influences the Ru nanoparticles, thereby improving their catalytic performance. This design produces a highly effective catalyst for hydrogen evolution reactions.

#### 2.1.2 Design principles for alkaline conditions

In alkaline conditions, the concentration of protons is minimal, necessitating the dissociation of water molecules (H_2_O → H_ad_ + OH^−^) to produce protons (Fig. 2).^62^ The dissociation of water is identified as the rate-limiting step in this process.^63^ The kinetics of the HER in alkaline solutions are slower compared to acidic conditions, primarily due to the greater energy required to break the H–OH bond.^64^ In spite of this kinetic limitation, alkaline systems present unique practical benefits for large-scale hydrogen production.^65^ The reduced corrosivity of alkaline solutions improves the electrochemical stability of electrode materials, leading to lower degradation rates for both catalysts and support structures. This stability is especially advantageous for continuous operation in industrial electrolysis systems, where long-term durability and low maintenance are essential factors.

**Fig. 3:**
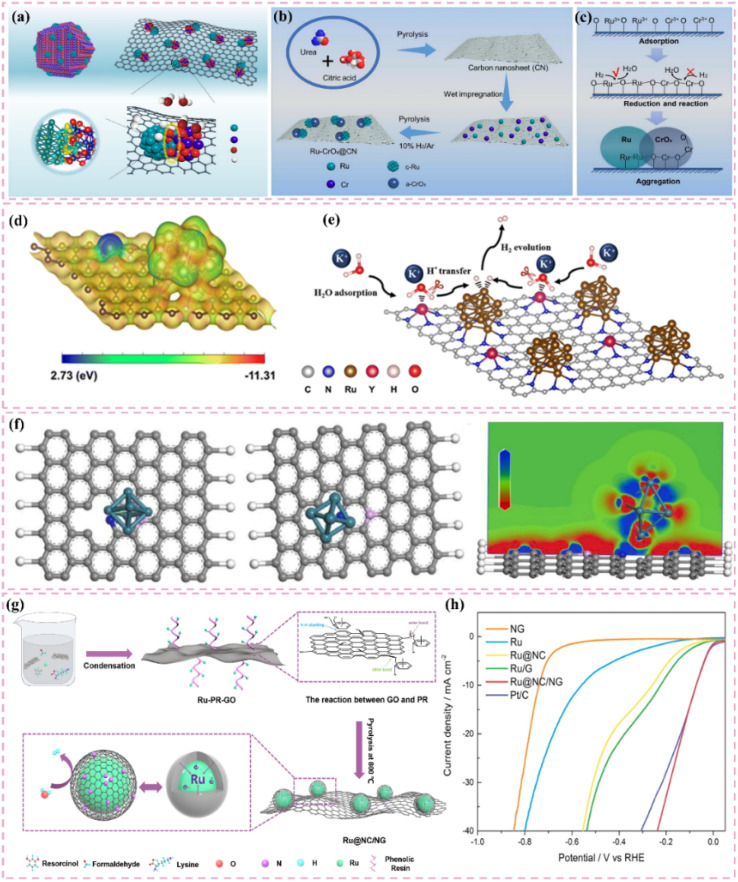
(a) A common supported catalyst and the cluster–cluster heterostructured catalyst. Scheme illustration of the synthesis route for Ru–CrO_*x*_@CN (b) and the proposed formation mechanism (c). Reproduced with permission from ref. 66. Copyright 2024, Springer Nature. (d) Calculated surface electrostatic potential distribution between Ru clusters and Y SAs in Ru–YNC. (e) Schematic diagram of cascade electrocatalysis mechanism for alkaline HER on Ru–YNC. Reproduced with permission from ref. 32. Copyright 2025, Royal Society of Chemistry. (f) Ru/d-NPC atomic structures before and after optimization of the calculation configuration, and electron localization function analysis for the first atomic layer. Reproduced with permission from ref. 71. Copyright 2022, Elsevier. (g) The fabrication scheme of Ru@NC/NG. (h) HER polarization curves 1.0 M PBS. Reproduced with permission from ref. 74. Copyright 2022, Elsevier.

The development of heterostructured electrocatalysts serves as a promising approach to improve the kinetics of alkaline HER. The construction of metal–metal oxide heterostructures on carbon supports allows for the exploitation of synergistic interface effects, which can enhance the catalytic performance for HER. For instance, it has been noted that cluster heterostructures consisting of crystalline Ru (c-Ru) clusters and amorphous CrO_*x*_ (a-CrO_*x*_) clusters on nitrogen-doped carbon nanosheets (CN) (CrO_*x*_@CN) can be synthesized (Fig. 3a).^66^ Due to the significant interactions between clusters, the Ru atoms within the Ru clusters can infiltrate the interface into CrO_*x*_. This phenomenon leads to improved interfacial charge redistribution and a higher adsorption energy of reaction intermediates, which can accelerate the rate-determining Volmer step of alkaline hydrogen electrocatalysis (Fig. 3b and c). Simultaneously, the strategic adjustment of electronic interactions among components within the heterostructure has demonstrated effectiveness in improving the adsorption and activation of reaction intermediates. For example, Zhang *et al.*^32^ created a heterostructured catalyst that incorporates Ru clusters and Y single atoms supported on N-doped carbon materials. This design utilizes the distinct benefits of Ru and Y species, spatially separating the water adsorption/dissociation and hydrogen adsorption processes while enhancing the electronic structure of the catalyst (Fig. 3d and e). This leads to outstanding performance in alkaline HER. Additionally, the design of carbon supports featuring hydrophilic surfaces and suitable pore structures can improve the adsorption and diffusion of water molecules, thereby facilitating water dissociation. This design approach enhances the overall efficiency of the HER process. Certain studies have detailed the preparation of Ru catalysts supported on carbon supports characterized by abundant mesoporous and macroporous structures.^36,67^ The porous structure of these catalysts facilitates water molecule transport and dissociation in alkaline media, thereby enhancing HER performance.

#### 2.1.3 Design principles for neutral conditions

The strategy to improve the performance of carbon-supported Ru-based catalysts in neutral conditions has a distinct emphasis. Acidic conditions focus on reducing strong acid corrosion and promoting effective proton adsorption.^68^ Alkaline conditions, conversely, facilitate the enhancement of hydrolysis.^69,70^ Neutral conditions present increased complexity due to the need to address both slow reaction kinetics and insufficient proton supply.

Improving intrinsic activity under neutral conditions is essential for optimizing catalyst performance. Doping with additional elements has proven effective alongside strategies like tuning the electronic structure and exposing active sites. Li *et al.*^71^ synthesized a catalyst (Ru/D-NPC) that consists of Ru nanoparticles interfacially coupled with defect-rich nitrogen- and phosphorus-*co*-doped carbon nanosheets. This catalyst demonstrated exceptional HER performance throughout the entire pH spectrum, especially under neutral conditions. The exceptional performance is ascribed to the Ru–N bond at the interface, which enhances the metal–support interaction and alters the electronic structure of Ru, thus optimizing the HER kinetics of the Ru active sites (Fig. 3f). Theoretical calculations supported this hypothesis.

Moreover, improving the hydrophilicity and water-splitting efficiency of catalysts is an essential approach. The synthesis of catalysts featuring a hydrophilic core–shell architecture has demonstrated an increase in hydrophilicity, facilitating the adsorption and diffusion of water molecules and enhancing their water-splitting efficiency. Certain carbon-supported Ru-based catalysts with core–shell architectures exhibit hydrophilic outer shells, which improve the accessibility and adsorption of water molecules under neutral conditions. In this context, the internal Ru nanoparticles act as active sites that enhance water cleavage and hydrogen production, thus optimizing the performance of HER in neutral media.^72,73^

Additionally, the choice of carbon supports that exhibit adequate chemical stability and corrosion resistance, along with suitable surface modification and catalyst protection methods, can improve the operational lifespan and stability of catalysts under neutral conditions. For example, the catalyst developed by Ma *et al.*,^74^ which consists of ultrafine Ru nanoparticles encapsulated on N-doped graphene sheets, exhibited remarkable stability in a neutral electrolyte. The stability was attained through various factors, notably the small size of the Ru nanoparticles and the stabilizing support along with the corrosion-resistant characteristics of the N-doped graphene (Fig. 3g). The catalyst exhibits significant activity in neutral media (Fig. 3h).

The development of carbon-supported Ru catalysts for hydrogen evolution across different reaction environments (acidic, alkaline, or neutral) should be guided by the fundamental reaction mechanisms and key design principles. The focus of these strategies should be on enhancing structural characteristics and ensuring the compatibility of active sites with the reaction environment to facilitate effective hydrogen evolution. An in-depth analysis of the design principles of catalysts across various media could create a theoretical framework and offer practical guidelines for developing high-performance and stable carbon-supported Ru-based catalysts.

### 2.2 Precise regulation of active sites

The optimization of HER performance has prompted the engineering of Ru active sites at different spatial scales, specifically as single atoms, clusters (ranging from a few to tens of atoms), and nanoparticles. A thorough comprehension of the trade-offs among these configurations is crucial for informed catalyst design. Single-atom catalysts optimize atom utilization efficiency and frequently demonstrate remarkable intrinsic activity as a result of their distinctive coordination environment. Nonetheless, they may experience restricted stability because of migration and agglomeration, potentially rendering them suboptimal for reactions that necessitate ensemble sites (*e.g.*, the Tafel step). Nanoparticles exhibit a high density of conventional active sites and typically demonstrate enhanced stability. However, their larger size results in reduced atom efficiency and an increased fraction of under-coordinated surface atoms with suboptimal adsorption energies. Clusters represent a strategic intermediary, providing a balance between the high atomic efficiency of single atoms and the strong catalytic characteristics of nanoparticles. They can promote synergistic effects and frequently display unique electronic structures that are different from both SAs and NPs. The selection among these options entails a fundamental trade-off involving atomic efficiency, stability, synthetic complexity, and appropriateness for reaction steps. Table 1 presents a comparative summary of these trade-offs.

**Table 1:** Comparison and trade-offs of single-atom, cluster, and nanoparticle Ru-based catalysts for HER

Characteristics	Single-atom	Cluster	Nanoparticle
Atomic utilization rate	Extremely high	High	Low to moderate
Intrinsic activity	Typically high	High	Moderate
Stability	Prone to migration and agglomeration	Superior to single atoms but inferior to nanoparticles	Typically highest
Active site type	Single, isolated site	Ensemble site, potentially exhibiting synergistic effects	Traditional surface site
Synthetic control difficulty	Requires strong anchor positioning points to prevent agglomeration	Controlling particle size and distribution uniformity	Relatively mature and straightforward
Primary advantages	Highest quality activity, well-defined structure–activity relationships	Balanced atom efficiency with activity/stability	Robust preparation and stability
Primary disadvantages	Stability issues, potential lack of ensemble sites	Challenging synthetic control	Low atom efficiency, high precious metal consumption

Recent studies demonstrate a strong correlation between the atomic-level configurations of Ru-based catalysts and their HER activity. The precise modulation of the coordination environment and electronic structure of active sites can enhance the adsorption behavior of reaction intermediates and address the performance limitations of traditional catalysts. The regulatory strategies are manifested at three primary levels. First, the single-atom dispersion strategy optimizes the use of active sites and adjusts the d-band center position, which in turn improves the efficiency and catalytic performance of these sites.^75,76^ Secondly, the cooperative interaction between nanoclusters and individual atoms enhance the development of delocalized-localized electronic structures.^77,78^ Thirdly, the design of a heterostructure influences the adsorption strength of key intermediates through interfacial charge redistribution effects, thereby optimizing the adsorption/desorption equilibrium during the reaction.^79,80^

The evolution of advanced synthesis technologies and *in situ* characterization has led to a shift from initial empirical exploration to a more systematic approach in the precise regulation of Ru active sites. This advancement has created a strong scientific basis for the development of the next generation of high-efficiency HER catalysts. Consequently, accurate modulation of the Ru active site microenvironment *via* approaches such as single-atom dispersion, nanocluster synergy, and heterostructure design has emerged as a crucial strategy to address the performance limitations.

#### 2.2.1 Optimizing coordination environments and electronic structures

The strategy of single-atom dispersion is essential for the accurate regulation of active sites. Dispersing metal atoms as individual entities on the support surface maximizes atom utilization and enhances catalytic performance, enabling each metal atom to act as an active site.^81–83^ In the Ru single-atom catalyst (Ru SAC) system, the introduction of heteroatoms such as N, B, and P into carbon supports generates a wealth of anchoring sites. The electronic state distribution of Ru is modified by these sites through coordination structures such as Ru–N_*x*_ and Ru–C_*x*_, which in turn enhances its intrinsic HER activity.^84,85^

**Fig. 4:**
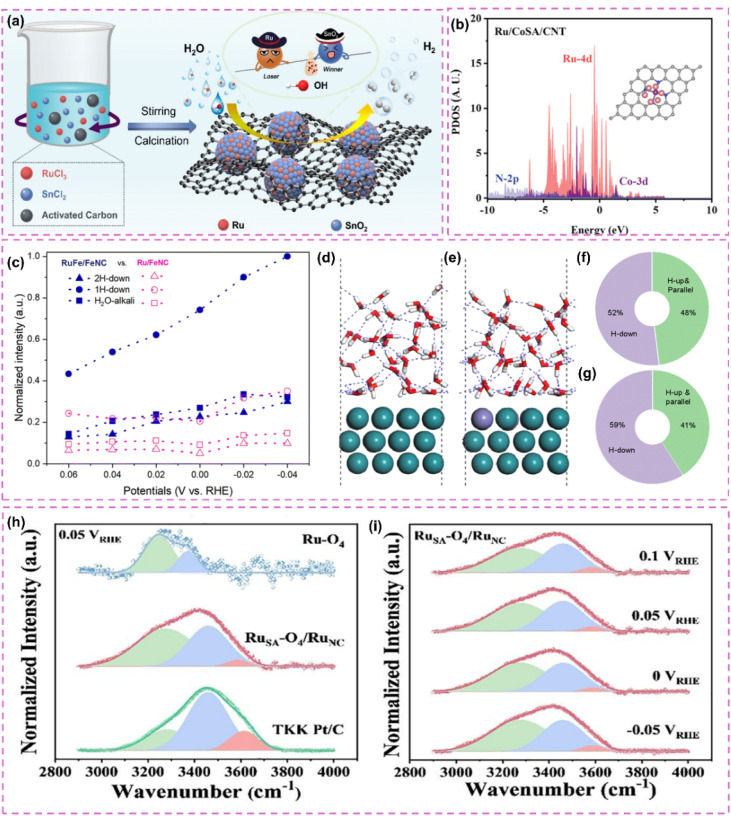
(a) Synthesis scheme of Ru SAs–SnO_2_/C. Reproduced with permission from ref. 86. Copyright 2022, Wiley-VCH. (b) Calculated density of states for Ru/Co_SA_/CNT. Reproduced with permission from ref. 87. Copyright 2025, Wiley-VCH. (c) The distribution of various water molecules evaluated by fitting the Raman peaks ranging from 3000 to 3800 cm^−1^. The water molecules are 2H-down, 1H-down, and H_2_O-alkali with wavenumbers from low to high. (d) and (e) the AIMD final snapshots of water distribution over single-atomic Fe modified and pure Ru surfaces. Statistical distribution results of interfacial one-layer water molecules on Ru (f) and RuFe (g) surfaces from AIMD simulations. Reproduced with permission from ref. 88. Copyright 2025, Royal Society of Chemistry. (h) *In situ* ATR-SEIRAS spectra of interfacial water on Ru_SA_–O_4_/Ru_NC_, Ru_SA_–O_4_, and Pt/C electrocatalysts at 0.05 V *vs.* RHE. (i) *In situ* ATR-SEIRAS spectra of interfacial water on Ru_SA_–O_4_/Ru_NC_. Reproduced with permission from ref. 89. Copyright 2024, Elsevier.

The catalytic properties of Ru single atoms significantly depend on their local coordination structures. In pursuit of effective electronic structure modulation, various investigations have thoroughly examined the impact of the competitive adsorption mechanism on the reaction pathway, thus providing innovative approaches for enhancing catalyst performance. Zhang *et al.*^86^ developed a Ru single-atom modified SnO_2_/C catalyst (Ru SAs–SnO_2_/C) and implemented a competitive adsorption strategy (Fig. 4a). Utilizing the pronounced affinity of SnO_2_ for hydroxyl groups (OH_ad_), competitive adsorption with Ru sites was established, which notably reduced the detrimental impact of OH_ad_ on Ru during alkaline HER. Calculations based on DFT indicated that SnO_2_ showed a preference for the adsorption of OH_ad_, which in turn expedited its desorption (OH_ad_ + e^−^ ⇌ OH^−^) during the Volmer step, thereby promoting the swift regeneration of Ru sites. The catalyst achieved a remarkably low overpotential of only 10 mV at a current density of 10 mA cm^−2^. Additionally, Wang *et al.*^87^ developed and created a Ru atom cluster (Ru/Co_SA_/CNT) catalyst that was modified with single-atom CoN_4_, leading to a notable improvement in alkaline HER performance *via* a dual regulatory mechanism. The CoN_4_ support demonstrated a significant ability to adsorb OH_ad_ and was capable of competing with Ru active sites for OH_ad_ adsorption, thus reducing the obstruction of Ru sites by OH_ad_. The CoN_4_ support influenced OH_ad_ behavior through competitive adsorption and enhanced the electronic structure of Ru atom clusters *via* electronic interactions (Fig. 4b), thereby further boosting the catalyst's performance. Furthermore, the electronic interactions between the CoN_4_ support and Ru atom clusters played a crucial role in preventing Ru atom migration and aggregation, thereby improving the structural stability of the catalyst.

Expanding on a comprehensive grasp of the coordination environment, the subsequent refinement of the electronic structure and interfacial microenvironment stands out as a crucial approach to improve catalyst performance. He *et al.*^88^ developed Fe-alloyed defective carbon-supported RuFe nanoparticles (RuFe/FeNC) using a sequential approach that involved catalytic polymerization, ion reduction, and pyrolysis. Fe doping optimized the intrinsic electronic structure of the Ru surface, resulting in electron-rich Ru sites and electron-deficient Fe sites. These changes improved the water activation and proton capture efficiency of the Ru sites, fostering a microenvironment rich in hydrogen ions, akin to that of an acid. Additionally, Fe sites regulated interfacial water molecule distribution, increasing H-down type molecules (Fig. 4c–g). This diminished the hydrogen-bonding network of the catalyst surface, enhancing the efficiency of water dissociation. The higher affinity of Fe sites for hydroxide ions (OH^−^) reduced the blocking of Ru sites induced by OH^−^. The adsorption of OH^−^ on Fe sites reduced the energy barrier for water dissociation on Ru sites. Furthermore, the accurate regulation of the spatial positioning and local microenvironment of metal single atoms on the support can optimize their electronic structure and interfacial microenvironment, thereby significantly improving the activity and stability of the catalyst. Subsequently, a different investigation introduced a dual active-site electrocatalyst (Ru_SA_–O_4_/Ru_NC_) that combines defective Ru nanoclusters with oxygen-coordinated Ru single atoms, showcasing exceptional HER performance *via* a distinctive synergistic mechanism.^89^ The interaction between oxygen-coordinated Ru single atoms (Ru_SA_–O_4_) and defective Ru nanoclusters (Ru_NC_) led to modulation of the electronic structure. Ru_SA_–O_4_, possessing a positive charge, aligned interfacial water molecules in an O-down (H_2_O↓) conformation (Fig. 4h and i). This improved the hydrogen-bonding network, promoting the Volmer step in alkaline hydrogen oxidation and hydrolysis in alkaline HER. The defective Ru nanoclusters predominantly facilitated hydrogen adsorption and dissociation, thereby inhibiting competitive adsorption among reaction intermediates.

Single-atom catalysts currently face challenges related to their long-term stability.^90^ These results are from the high-energy Ru single atoms on the surface being prone to structural reconfiguration during the reaction, leading to a decline in activity. Consequently, researchers are committed to investigating various strategies to improve the stability of single-atom catalysts. Among these approaches, careful engineering of the electronic structure and geometric configuration of the support can effectively stabilize Ru single atoms, thus minimizing their migration and aggregation under reaction conditions.^91–93^ Additionally, the construction of dual-active sites or multifunctional synergistic catalytic networks can mitigate stress and reduce the energy levels of single-atom sites, thereby enhancing their stability.^94,95^ These approaches provide new insights and pathways for the development of high-performance and durable single-atom catalysts.

The single-atom dispersion strategy has significantly improved the HER performance of Ru-based catalysts through precise modulation of the coordination environment and electronic structure of the active sites. The precise regulation of coordination environments significantly influences the electronic structure of Ru sites. This process is essential for adjusting the adsorption strength of reaction intermediates. Coupling Ru with more electronegative atoms results in the withdrawal of electron density from the Ru center, thereby lowering its d-band center. This procedure reduces the strength of the metal–H bond, thereby optimizing the free energy of hydrogen adsorption to near thermo-neutrality. Subtle alterations in the first and second coordination layers effectively influence catalytic activity by directly affecting reaction energetics.

The ongoing advancements in synthesis technology and the enhanced understanding of support effects suggest that single-atom dispersed Ru-based catalysts will increasingly contribute to efficient, stable, and cost-effective hydrogen evolution. Future research should focus on enhancing the stability of single atoms and investigating simpler, more efficient, and cost-effective synthesis methods to facilitate the broader application of single-atom dispersed Ru-based catalysts in practical water electrolysis devices. Two primary challenges hinder the industrialization of single-atom catalysts. Long-term stability is a critical issue, as high-surface-energy single atoms are prone to migration and aggregation under reaction conditions. Achieving significant macroscopic current output necessitates high metal loading, a challenge when aiming to preserve single-atom dispersion. Numerous high-performance single-atom catalysts documented in the literature depend on intricate multi-step syntheses or costly supports, which present significant obstacles to large-scale production due to their complexity and expense. When assessing single-atom catalysts, it is crucial to extend the evaluation beyond intrinsic activity to include stability, loading, and the economic aspects of the synthetic process. Therefore, the design of supports with robust anchoring and mechanical stability is essential for single-atom catalysts to realize practical applications, as these features can mitigate activity loss while preserving high activity.

#### 2.2.2 Enhancing electron coupling and mass transfer

Traditional research has focused on the development of individual active sites. However, the limited hydrolysis dissociation capability of single-atom sites often restricts effective HER promotion. In the interim, although simple nanoclusters exhibit certain activity, they are susceptible to aggregation, which diminishes active sites and undermines catalytic performance. In recent years, the development of a “single-atom-nanocluster” dual-site system has provided an innovative approach for the design of Ru-based catalysts.^70,96,97^ Single atoms and nanoclusters interact synergistically through precise regulation. This improves hydrolysis dissociation and optimizes hydrogen adsorption kinetics through electronic coupling effects, addressing the limitations of single active sites and enhancing catalytic performance.

Numerous studies indicate that the rational engineering of the support's surface structure can effectively mitigate the agglomeration of nanoclusters and the loss of single atoms.^59,98,99^ This allows the catalyst to sustain its long-term activity under severe reaction conditions. For example, Liu *et al.*^92^ demonstrated that optimizing the geometry of supports can significantly enhance the electronic properties and stability of single-atom catalysts, thereby improving catalytic performance. The researchers achieved efficient dispersion and stabilization of single-atom Ru sites by preparing B, N co-doped carbon-supported Ru catalysts (RuBNC2000) with ultra-high curvature. The strain effect of the curved support induces a 1.5% compression of the Ru–N bond and a 4% stretching of the Ru–B bond, leading to the accumulation of positive charges at the Ru center and the elimination of spin polarization. The modulation of the electronic structure enhances the adsorption strength of the H* intermediate, allowing the catalyst to demonstrate intrinsic activity and superior stability compared to commercial Pt/C in alkaline media. The analysis integrating experimental and theoretical calculations indicates that curved surface supports offer a novel approach to modulate catalytic performance through alterations in bond lengths between monoatomic metal centers and adjacent ligand heteroatoms. This study confirms the significant influence of the support surface structure design on catalyst performance and offers a novel approach for developing low-cost, efficient, and stable single-atom catalysts. By accurately controlling the geometrical arrangement of the supports, the electronic environment of the single-atom active sites can be optimized, thereby significantly improving the potential applications of the catalysts in energy conversion reactions.

The dual-site system of “single-atom-nanocluster” exhibits a significant synergistic interaction between single atoms and nanoclusters, particularly in electron transfer processes and the distribution of active site functions. Electron transfer influences the electronic characteristics of individual atoms, consequently modifying their adsorption capacity and catalytic activity in relation to reactants. The division of active site responsibilities allows the catalyst to sustain high efficiency throughout various reaction steps. The nitrogen-doped mesoporous carbon-supported Ru single-atom and nanocluster catalyst (NMC-Ru_SA+NC_) exhibits electronic transfer from Ru nanoclusters to single-atom sites, resulting in an increased electron density around the single-atom sites.^100^ This phenomenon influences the adsorption capacity and catalytic activity of individual atoms in relation to reactants. This electronic synergy optimizes the adsorption energy of reaction intermediates, reduces the activation energy of reactions, and improves HER performance.

**Fig. 5:**
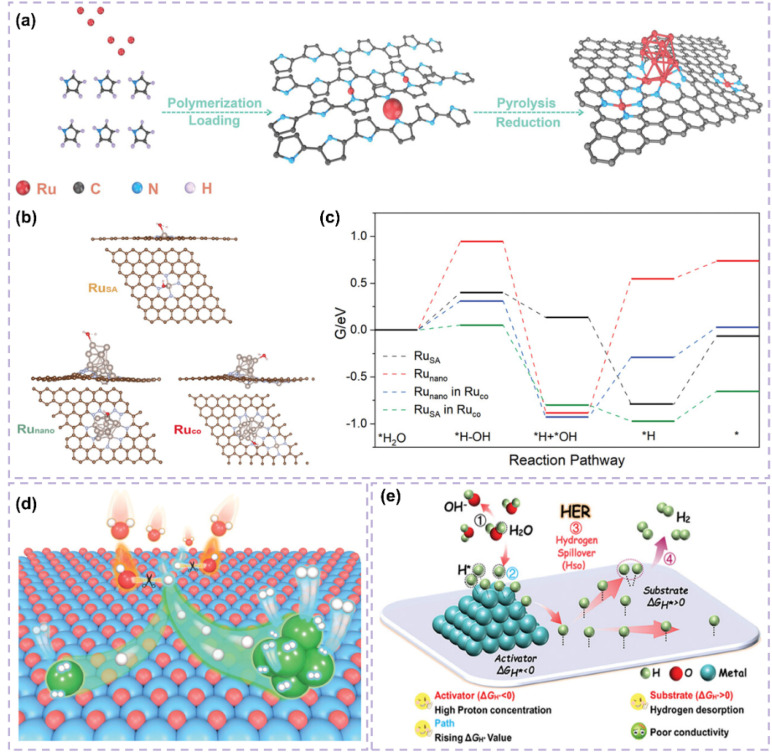
(a) Schemetic synthesis of Ru_1,*n*_–NC. (b) The transition states of H_2_O activation over different models. (c) Free energy diagrams of the elementary steps in alkaline HER for bare Ru_nano_, bare Ru_SA_, and Ru_co_. Reproduced with permission from ref. 102. Copyright 2022, Wiley-VCH. (d) Schematic diagram illustrating the role of each component in the Ru/NC when catalyzing the alkaline HER. Reproduced with permission from ref. 99. Copyright 2023, Wiley-VCH. (e) The theoretical insights of hydrogen spillover in the HER. Reproduced with permission from ref. 109. Copyright 2024, Wiley-VCH.

Engineering carbon supports through defect introduction or tailored pore structuring can improve their specific surface area and optimize the dispersion and exposure of active sites.^101–104^ Furthermore, the refinement of pore structure enhances reactant adsorption and diffusion, reduces mass-transfer resistance, and consequently increases the catalytic efficiency and stability of the catalyst. He *et al.*^102^ developed an efficient base HER catalyst (Ru_1,*n*_-NC) by depositing dispersed Ru nanoparticles and adjacent single-atomic Ru onto a carbon support (Fig. 5a). This structure enhances active-site dispersion and improves reactant adsorption and diffusion through defective sites, resulting in an ultralow overpotential of 14.8 mV and a high turnover frequency of 1.25 H_2_ s^−1^, exceeding the performance of commercial Pt-carbon catalysts. This study, through experimental and theoretical analyses, demonstrated the substantial influence of support defects and pore structures on catalytic performance, reinforcing the notion that optimizing support structure can enhance catalyst activity and stability (Fig. 5b and c). In the HER process, single-atom and nanocluster sites exhibit different functionalities. Single-atom sites exhibit significant intrinsic activity and are capable of efficiently adsorbing and activating hydrogen atoms. Nanoclusters can function as active centers for hydrolysis dissociation, increasing the number of adsorption sites and facilitating the dissociation of water molecules (Fig. 5d).^99^ The interaction between these two site types enables the catalyst to sustain its efficiency across various reaction steps, thus improving its overall catalytic performance.

#### 2.2.3 Heterogeneous structures enhancing catalytic performance

The heterostructure design effectively regulates the adsorption behaviors of key intermediates, optimizing catalytic performance in a targeted way. It also facilitates the creation of robust electronic interaction interfaces between Ru and components like metal oxides, nitrides, and carbon matrices, allowing for precise control over the adsorption behaviors of crucial intermediates (H, OH, H_2_O*) to enhance catalyst performance across various reaction scenarios.^105–107^

The investigation into carbon-supported Ru-based catalysts reveals that heterogeneous structure design presents considerable potential for enhancing catalytic performance. The hydrogen spillover mechanism, recognized as a significant synergistic effect, plays a distinct role in heterostructures. The phenomenon of hydrogen spillover refers to the movement of hydrogen atoms from metallic sites to catalyst supports or other metallic surfaces.^108,109^ This process optimizes the adsorption, dissociation, and recombination of hydrogen, significantly improving the performance of the catalyst.^19^ The development of carbon-supported Ru-based catalysts featuring a heterogeneous structure facilitates optimal metal–support interactions, which promote effective hydrogen spillover and allow for the efficient migration of hydrogen atoms across various material interfaces. This mechanism enhances the catalyst's activity and stability while broadening its potential applications across diverse reaction environments. Hydrogen spillover provides a plausible rationale for the improved HER performance observed in heterostructure catalysts. However, its precise role in catalysis continues to be a subject of debate. The primary concern is to differentiate whether performance improvements arise from hydrogen atom migration or from interface electronic effects, such as charge transfer from the metal to the support, which enhances H* adsorption energy. Therefore, asserting that hydrogen spillover is the primary factor necessitates a variety of evidence beyond mere high performance. Comparing the performance of physical mixtures and intimate heterostructures may provide initial evidence of interface dependence. Spillover can kinetically act as a rate-determining step within certain overpotential ranges, particularly at high overpotentials, as indicated by notable alterations in the Tafel slope. Theoretically, DFT calculations are required to verify that the energy barrier for hydrogen atom migration across the interface is lower than that for direct recombination. Direct experimental investigation of hydrogen species on non-metal supports, such as through *in situ* spectroscopy, offers significant validation. Chen's work^109^ illustrates that encapsulating ultrafine Ru and MoO_2_ nanoparticles within nitrogen-doped carbon fibers (NCF) results in the formation of a Ru/MoO_2_ heterostructure characterized by a Ru–O–Mo bridging configuration. This configuration improves electron transfer and promotes the hydrogen spillover phenomenon. In this context, hydrogen spillover is associated with electron transfer from Ru to MoO_2_, which inherently modifies Ru's electronic structure and enhances HER activity (Fig. 5e). The exceptional performance is likely attributable to the synergistic interaction between interfacial electronic effects and potential hydrogen spillover, rather than a singular mechanism. Future research must systematically verify the role of hydrogen spillover in the HER using the previously mentioned multi-criterion framework to resolve disputes and confirm its true significance.

**Fig. 6:**
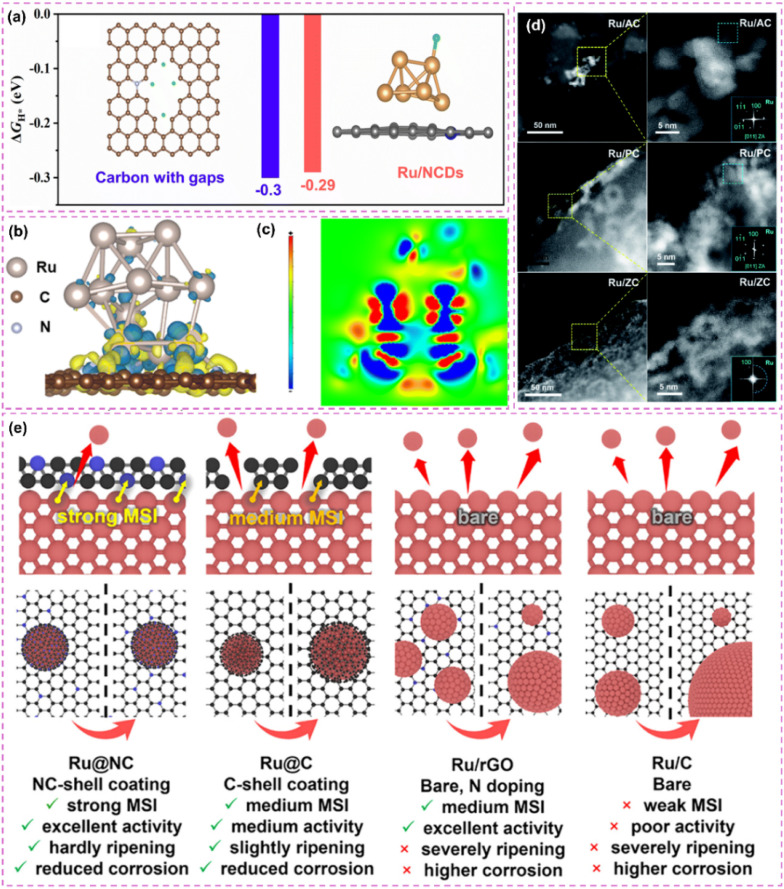
(a) Calculated free energy diagram for carbon with gaps and Ru/NCDs. Reproduced with permission from ref. 110. Copyright 2024, Royal Society of Chemistry. (b) Model of c-Ru@NPC with charge density difference and (c) corresponding two-dimensional slice. Reproduced with permission from ref. 10. Copyright 2025, Royal Society of Chemistry. (d) HAADF-STEM images of Ru/AC, Ru/PC, and Ru/ZC catalysts. Reproduced with permission from ref. 112. Copyright 2022, Royal Society of Chemistry. (e) Schematic illustration of the mechanism of N doping and carbon shell structure in stabilizing Ru NPs. Reproduced with permission from ref. 113. Copyright 2025, Wiley-VCH.

Improving the interfacial area for hydrogen spillover presents a valuable approach to increasing the catalytic activity of carbon-supported Ru-based catalysts, as hydrogen spillover occurs at the interface. Liu *et al.*^110^ investigated the pivotal role of carbon supports in N-doped carbon dots-confined Ru nanoparticle catalysts (Ru/NCDs). The domain-limiting effect of carbon dots effectively confines Ru nanoparticles within the carbon support, reducing their size. The ultra-small Ru nanoparticles enhance interaction with the carbon support, resulting in a greater number of Ru-carbon interfaces and creating numerous hydrogen spillover pathways. Their reduced dimensions further reveal a higher number of active sites, thereby improving catalyst efficiency for hydrogen adsorption and desorption. Furthermore, the carbon support creates multiple hydrogen adsorption sites resembling Ru due to domain-limiting effects. These sites effectively capture hydrogen atoms, functioning as a “supply station” for hydrogen spillover and consistently providing hydrogen to Ru particles (Fig. 6a). This enhances the efficiency of the hydrogen spillover process. The interaction between the Ru-like characteristics of the carbon support and the domain-limiting effect enhances the catalyst's performance, resulting in superior hydrogenation activity and stability in acidic and basic solutions.

The synergistic modulation of electronic structures, in addition to optimizing active sites, enhances catalytic performance. The interaction between metal and support can modify electron distribution, consequently affecting catalytic activity. The interaction between N-doped carbon supports and Ru modifies the electronic structure of Ru, which in turn facilitates hydrogen adsorption and desorption. The investigation conducted by Li *et al.*^10^ provides a comprehensive examination of N-doped hollow porous carbon-supported Ru cluster catalysts (c-Ru@H-NPC), highlighting the relationship between N-doped carbon supports and Ru. The findings indicate that nitrogen doping enhances the hybridization of d–p orbitals between Ru and carbon, optimizes the pathways for electron transfer, and alters the electronic structure of the active sites (Fig. 6b and c). This interaction lowers the adsorption energy of hydrogen intermediates and improves the adsorption of water molecules, thereby speeding up the Volmer reaction. Additionally, the hollow structure offers more active sites and promotes swift hydrogen diffusion, further boosting the overall performance of the catalyst.

Elemental doping serves as a crucial approach for adjusting the electronic structure and catalytic efficiency of materials through modifications in electron distribution and the geometric arrangement surrounding active sites. In catalyst design, the incorporation of heteroatoms such as nitrogen, boron, and phosphorus into carbon supports or metal active centers facilitates meticulous control over electron cloud density and energy band structure. This optimization modifies the adsorption strength of reaction intermediates and enhances reaction pathways. For example, the electronic microenvironment of Ru-based catalysts was adjusted through the co-doping of boron and nitrogen into the carbon matrix.^111^ These changes notably improved their HER performance over a wide pH range. The effect of boron on electron modulation enhances the processes of hydrogen adsorption and water activation through the adjustment of electron distribution at active sites. The catalyst demonstrates remarkable activity and stability across acidic, neutral, and basic environments.

Electronic-structure synergy enhances catalytic performance by leveraging internal electronic properties, whereas a design that emphasizes interfacial interactions focuses on the characteristics of heterostructure interfaces. Building metal–carbon heterostructures utilizes the differences in interfacial energy levels and charge transfer to efficiently adjust the electronic structure. Jiang *et al.*^112^ performed an in-depth investigation into the interfacial interactions between Ru and different carbon supports to improve HER catalytic performance. The study examined the effects of amorphous carbon (AC), porous carbon (PC), and metal–organic framework-derived carbon (ZC) supports on Ru species (Fig. 6d). The findings indicate that ZC supports enhance metal–support interactions, keeping Ru in a highly dispersed metallic form, which in turn boosts HER activity and stability. Furthermore, acid etching treatment (AET) was utilized to enhance interfacial properties. This treatment significantly decreased Ru particle size and enhanced the exposed Ru–C interface. The optimized Ru/ZC-E50 catalyst exhibited a remarkably low overpotential of 29 mV at 10 mA cm^−2^, along with outstanding stability in alkaline media. This study presents a new approach for the design of high-efficiency HER catalysts. The design of core–shell structures affects electron transfer and reactivity through interfacial interactions. Geng *et al.*^113^ demonstrated the effectiveness of the core–shell structure in their study. The Ru@NC catalyst exhibits a robust metal–support interaction between nitrogen-doped carbon shells and Ru nanoparticles. This interaction promotes electron transfer from Ru nanoparticles to carbon shells, modifying the electronic binding energy of the Ru nanoparticles. As a result, the electronic structure of the catalyst is optimized, leading to improved hydrogen adsorption and desorption capabilities, which enhances HER activity. The interaction enhances the chemical stability and corrosion resistance of the catalyst (Fig. 6e).

Interfacial interactions improve performance mainly by facilitating electron transfer. Additionally, enhancing mass and charge transport significantly improves catalytic efficiency in matter and charge transport processes. Designing a porous structure enhances the specific surface area of the catalyst, increases the number of active sites and mass-transfer channels, and promotes the diffusion processes of reactants and products. This design concept provides substantial theoretical insights and practical guidance for developing highly efficient catalysts. Ma *et al.*^59^ synthesized a Ru-based catalyst that incorporates Ru nanoclusters and Ru single atoms co-embedded within nitrogen-doped carbon spheres (Ru/NPCS). The porous structure significantly enhances the catalyst's specific surface area by generating additional surface sites, which increases active sites and improves the adsorption of reactant molecules and their reaction opportunities. Furthermore, the presence of pores and channels within the structure facilitates the transport of reactants and products, minimizes diffusion resistance, speeds up reaction kinetics, and boosts catalytic efficiency. The experimental findings demonstrate that this catalyst outperforms commercial Pt catalysts in terms of activity in acidic environments, while also showcasing remarkable mass activity and stability.

Improving the conductivity of carbon supports facilitates electron transport and reduces charge transfer resistance. The use of highly conductive carbon supports, including carbon nanotubes and graphene, greatly enhances charge transfer efficiency. Ren *et al.*^114^ created a triatomic Ru-based catalyst (Ru_3_/OCNT) using a rapid pyrolysis precursor strategy, achieving a homogeneous dispersion on oxidized carbon nanotubes. This catalyst demonstrated superior performance in the alkaline hydrogen evolution reaction, characterized by low overpotential, high specific activity, and remarkable stability. The robust carbon–metal interaction contributed to enhanced electron transport efficiency and optimized the geometric and electronic structures of the metal active sites. These improvements led to a notable decrease in hydrogen adsorption energy and facilitated hydrogen desorption.

The design of heterostructures in carbon-supported Ru-based catalysts demonstrates significant potential for enhancing hydrogen evolution performance through a range of improvement mechanisms, including active site optimization, electronic structure synergy, enhancement of interfacial interactions, hydrogen spillover mechanisms, and optimization of mass and charge transport. Despite the significant advancements achieved in recent studies, obstacles like elevated preparation expenses, the feasibility of large-scale production, and long-term stability in real-world applications continue to pose challenges. Future investigations should expand on the design principles and synthesis methods of heterostructures, integrating both theoretical and experimental approaches. This will aid in the development of more efficient, stable, and cost-effective carbon-supported Ru-based catalysts, thereby facilitating the commercialization of hydrogen production technology through water electrolysis.

The design of heterostructures enhances performance but also increases synthetic complexity considerably. Requirements such as multi-step synthesis and precise interfacial control result in complex and costly processes that exhibit poor batch reproducibility, which contradicts the simplicity, stability, and efficiency necessary for industrial production. Moreover, numerous heterostructure catalysts face challenges in sustaining performance benefits at reduced Ru loadings. The pursuit of high performance through elevated Ru loadings compromises their economic competitiveness compared to Pt. Future research should concentrate on creating straightforward, scalable techniques for heterostructure preparation, as well as assessing their effectiveness and durability at low loadings and in challenging industrial environments.

## 3 Optimization of carbon supports and synergistic effects

In Ru-based catalysts, carbon supports function as active components rather than merely serving as inert support platforms. The interplay between their physical structure and chemical properties directly affects the electrical environment of the active sites, mass-transfer efficiency, and interfacial reaction kinetics.^115^ Carbon supports are essential for stabilizing the active metal Ru and significantly influence the catalyst's electronic structure, the distribution of active sites, the efficiency of reactant and product diffusion, and overall stability. Different types of carbon supports, due to their unique structural and performance characteristics, significantly interact with Ru-based active components, thereby affecting the catalytic efficiency of the HER. A thorough analysis of carbon support types and their characteristics is crucial for understanding the structure–activity relationship of catalysts, improving catalytic performance, and creating new and efficient catalysts.

### 3.1 Categories and characteristics of carbon supports

The role of carbon supports is essential in carbon-supported Ru-based catalysts, with their type and structure significantly influencing catalyst performance. Carbon materials exhibiting various structures possess distinct physical and chemical properties that influence their behavior in catalytic applications and the mechanisms underlying the enhancement of catalytic performance.^116^

Carbon materials exhibit distinct advantages based on their dimensional characteristics. The future design trend will likely progress towards multi-dimensional composites and functional integration. For example, the synergistic integration of surface functional groups from zero-dimensional carbon dots, the conductive network of one-dimensional carbon nanotubes, the high specific surface area of two-dimensional graphene, and the hierarchical pore structure of three-dimensional porous carbon can result in an effect where “1 + 1 > 2.” The controllable synthesis and large-scale production of complex structures continue to pose considerable challenges in the field of materials science.

#### 3.1.1 Mechanisms underlying the enhancement of catalytic performance by various structural carbon materials

The dimensional topology of carbon materials significantly influences catalytic performance by modulating electron conduction pathways, active site accessibility, and the kinetics of reaction mass transfer.^117^ The distinct physicochemical properties of each dimension of carbon materials influence their behavior in catalysts and the mechanisms of catalytic enhancement. Recent studies demonstrate that the structural design of carbon materials can address the activity and stability limitations of Ru-based catalysts. The mechanism encompasses various components, including electronic structure optimization, interfacial engineering, and microenvironmental modulation.^118^

**Fig. 7:**
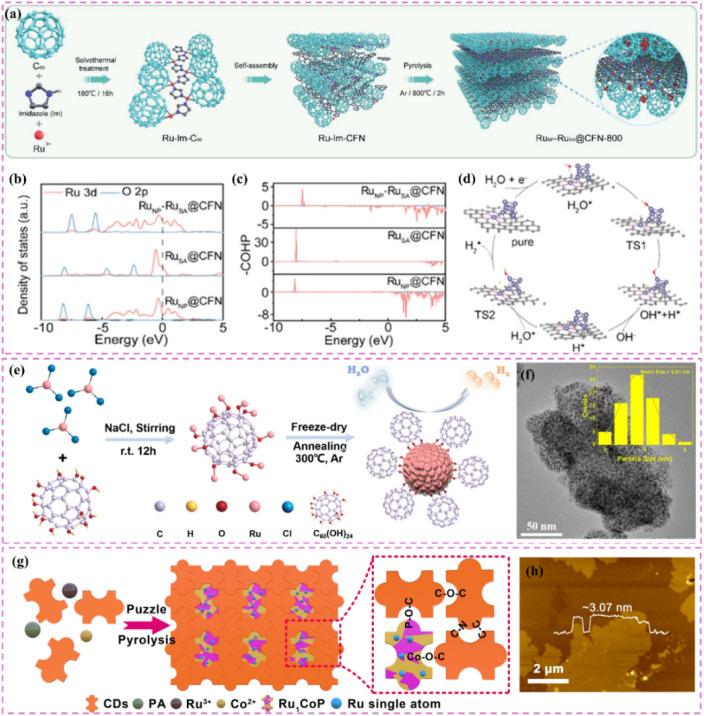
(a) Schematic illustration of the synthetic process of Ru_NP_-Ru_SA_@CFN-800. (b) DOS and (c) COHP diagram for Ru_NP_-Ru_SA_@CFN, Ru_SA_@CFN and Ru_NP_@CFN. (d) Proposed possible HER mechanism for Ru_NP_-Ru_SA_@CFN in alkaline medium. Reproduced with permission from ref. 120. Copyright 2023, Wiley-VCH. (e) Synthesis Procedure for Ru–OC_60_-300. TEM images of Ru–OC_60_-300. The inset in (f) shows the particle size distribution of Ru NPs. Reproduced with permission from ref. 121. Copyright 2023, American Chemical Society. (g) Formation of Ru_1_CoP/CDs. (h) AFM image of Ru_1_CoP/CDs-1000. Reproduced with permission from ref. 122. Copyright 2021, Wiley-VCH.

Zero-dimensional carbon materials, including fullerenes and carbon nanodots, exhibit distinctive physical and chemical properties that demonstrate significant promise in carbon-supported Ru-based catalysts.^119^ Fullerenes possess a cage-like architecture, and their significant specific surface area along with numerous chemically active sites render them excellent candidates for catalyst supports. Luo *et al.*^120^ devised a lattice-confined *in situ* reduction technique to synthesize the Ru_NP_-Ru_SA_@CFN-800 catalyst, which encapsulates Ru nanoparticles and single atoms within a three-dimensional crystalline fullerene framework (Fig. 7a). The unique lattice structure of fullerene effectively dispersed Ru species, thereby preventing particle aggregation and the deactivation of active sites. The interaction between fullerene and Ru species modified charge distribution and reduced the Gibbs free energy of intermediates in the hydrolysis dissociation process, thus enhancing hydrogen evolution kinetics (Fig. 7b–d). This study illustrates the potential of fullerenes as catalyst supports and offers new perspectives on the interaction mechanisms between fullerenes and active species through integrated experimental and theoretical analyses. Moreover, the significant reactivity of fullerenes can work in conjunction with Ru nanoparticles to facilitate electron transfer and the adsorption of reactants, thereby improving the overall efficacy of the catalyst. Li *et al.*^121^ utilized C_60_ fullerol (C_60_(OH)_24_) for the stabilization, dispersion, and activation of Ru nanoparticles (Fig. 7e). Particles connected through Ru–O–C_60_ bonds facilitate efficient alkaline HER. The electron-attracting properties of C_60_ fullerols significantly influence electron transfer from Ru to C_60_, thus enhancing intermediate adsorption characteristics. The enhanced electronic metal–support interaction (EMSI) significantly modifies the electronic structure of Ru nanoparticles, reduces the strength of Ru–H bonds, and decreases the dissociation energy of H_2_O. The cage-like architecture of C_60_ fullerol spatially restricts Ru nanoparticles, facilitating uniform distribution on the carbon support, even at elevated Ru loadings of 38%. Despite high loadings, the nanoparticles retain a small average size of 2.81 nm, which fully exposes active sites and facilitates efficient catalysis (Fig. 7f). The structural advantage of C_60_ fullerol renders it highly promising for the development of high-performance catalysts. In addition, carbon nanodots (CDs) exhibit a quantum size effect and possess numerous surface functional groups, enabling them to establish strong interactions with Ru nanoparticles and alter their electronic structure, thereby enhancing catalytic activity. Song *et al.*^122^ constructed single-atom Ru-doped CoP/CDs nanosheets by splicing CDs, demonstrating that the abundant surface functional groups of CDs function as jigsaw puzzle pieces, providing high-density binding sites for immobilized Ru single-atom sites (Fig. 7g and h). This design enhances the stability and conductivity of the catalyst while significantly improving the catalytic performance of HER by reducing the energy barrier for proton-coupled electron transfer and promoting the formation of H–H bonds *via* the incorporation of Ru single-atom sites. This study presents compelling evidence for the precise modulation of the electronic structure of metal active sites through the surface chemistry of CDs. It further illustrates the significant potential of the composite of CDs and metal nanoparticles in improving catalytic performance. In a similar vein, another study conducted by the group highlighted the essential function of CDs within the catalytic system.^123^ The authors highlighted that the small size and distinctive electron transfer capability of CDs render them excellent nanotemplates for regulating the growth and crystallization of CoRu nano-alloys while also inhibiting the aggregation of metal particles. Furthermore, the surface of CDs is abundant in functional groups that contain oxygen and nitrogen, which not only strengthen the interaction between the metal and carbon supports but also refine the electron transfer pathways, thereby greatly enhancing the stability and conductivity of the catalysts. This study illustrates the distinctive benefits of CDs for modulating the electronic structure of metals and enhancing catalytic performance, emphasizing their essential role in developing effective catalytic systems.

**Fig. 8:**
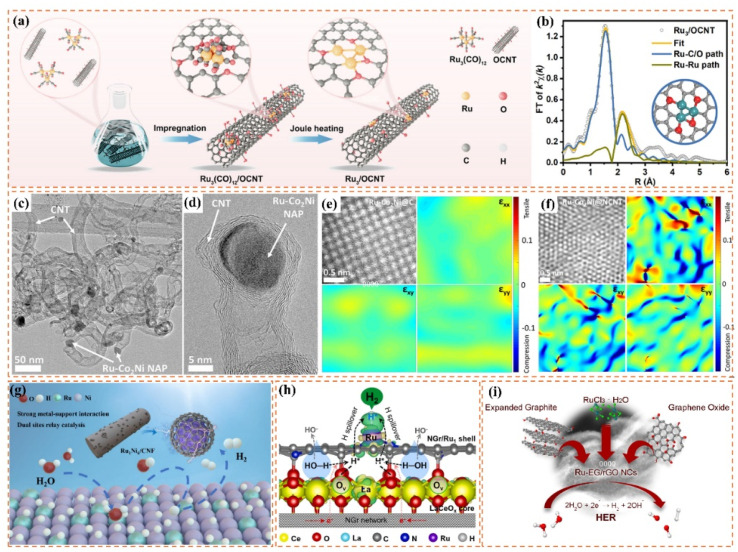
(a) Schematic diagram of the synthesis of Ru_3_/OCNT catalyst. (b) Ru_3_/OCNT and d) Ru_1_/OCNT at R space. Inset: the schematic model of Ru_3_/OCNT and Ru_1_/OCNT. C: grey, O: red, and Ru: green. Reproduced with permission from ref. 114. Copyright 2025, Wiley-VCH. (c and d) HRTEM images of the Ru–Co_2_Ni@NCNT. Atomic resolution image along with the *ε*_*xx*_, *ε*_*xy*,_*ε*_*yy*_ strain components determined *via* GPA of the (e) Ru–Co_2_Ni@C and (f) Ru–Co_2_Ni@NCNT. Reproduced with permission from ref. 125. Copyright 2025, Wiley-VCH. (g) Mechanism for enhancing the performance of Ru_1_Ni_6_/CNF. Reproduced with permission from ref. 126. Copyright 2025, Elsevier. (h) Possible hydrogen spillover mechanism for the alkaline HER over an advanced binary-site LaCeO_*x*_@NGr/Ru_1_ catalyst. Reproduced with permission from ref. 128. Copyright 2024, Elsevier. (i) Mechanisms for Enhancing the Performance of Ru/EG-rGO NCs. Reproduced with permission from ref. 129. Copyright 2024, American Chemical Society.

One-dimensional carbon materials, including carbon nanotubes, exhibit a tubular structure characterized by a high specific surface area, outstanding electrical conductivity, and remarkable mechanical strength.^124^ The tubular structure offers numerous pores and surface active sites that facilitate the dispersion and loading of Ru nanoparticles. Additionally, it enhances electron transfer efficiency and boosts the electrocatalytic activity of the catalysts due to its superior electrical conductivity. Ren *et al.*^114^ presented a triatomic Ru-based catalyst (Ru_3_/OCNT) utilizing oxidized carbon nanotubes (OCNT), which facilitated the uniform dispersion of Ru nanoclusters on carbon supports through a rapid pyrolysis precursor strategy (Fig. 8a). The tubular structure of OCNT offers numerous anchoring sites for Ru clusters, while its surface oxygen-containing functional groups enhance the electronic structure of Ru *via* strong metal–support interactions (SMSI) (Fig. 8b). The findings demonstrate that triatomic Ru clusters interact *via* metal–metal and metal–support bonding, resulting in distinct geometrical configurations and electronic state density distributions. DFT calculations indicate that the OCNT support lowers the hydrogen adsorption energy barrier and enhances H* desorption kinetics by altering the d-band center position of Ru. This modification allows Ru_3_/OCNT to achieve a HER catalytic efficiency near the thermodynamic limit in alkaline media. This study presents a novel approach to atomic-level structure modulation for designing one-dimensional carbon material-loaded metal cluster catalysts, and confirms the critical role of tubular carbon supports in enhancing the geometrical configuration and electronic structure of noble metal active sites. Despite significant advancements in the structural modulation of one-dimensional carbon material-loaded metal catalysts, optimizing the electronic structure and geometrical configuration of the active sites continues to be a critical challenge for improving catalytic performance. Building on this foundation, Majumdar *et al.*^125^ investigated the capabilities of one-dimensional carbon materials in electrocatalysis, specifically focusing on the optimization of Ru–Co_2_Ni nano-alloys and their encapsulation within nitrogen-doped carbon nanotubes to enhance efficiency in alkaline and acidic water electrolysis *via* strain engineering (Fig. 8c and d). The core–shell structure effectively inhibited the agglomeration of metal nanoparticles due to the encapsulation effect of highly graphitized carbon nanotubes and introduced compressive strains that significantly altered the electronic structure of the metal active sites (Fig. 8e and f). DFT calculations indicated that the incorporation of Ru enhanced the hydrogen adsorption energies at Co and Ni sites, while the strain effect of carbon nanotubes further facilitated hydrolysis dissociation and hydrogen release kinetics. This study validates the role of tubular carbon materials in modulating the electronic structure and geometrical configuration of metal nano-alloys and proposes a new strategy to optimize the active centers of noble and transition metals through strain engineering. Much like carbon nanotubes, carbon fibers, a significant carbon material, exhibit considerable promise in the realm of electrocatalysis. Huang *et al.*^126^ synthesized Ru-doped Ni nanoparticles encapsulated in graphitic carbon shells and supported on carbon nanofibers (Ru_1_Ni_6_/CNF) through electrospinning and pyrolysis techniques. The carbon fibers offered one-dimensional structural support while also enhancing the catalysts' performance due to their high specific surface area and outstanding electrical conductivity. The strong interaction between the graphitic carbon shells on the carbon fibers and the metal nanoparticles effectively prevented the agglomeration of the nanoparticles, safeguarded the catalyst from losing specific surface area due to thermal agglomeration, and enhanced charge transfer. The structural stability of the carbon fibers also played a crucial role in safeguarding the catalyst surface against electrochemical corrosion, leading to notable enhancements in both the stability and activity of the catalyst (Fig. 8g). DFT calculations indicated that the Ru sites exhibited significant adsorption and desorption efficiencies of water, which is crucial for the hydrolysis step in alkaline HER, whereas the Ni sites facilitated the recombination of hydrogen. The efficacy of this two-site grafted catalyst matches that of commercial noble metal catalysts, offering a novel perspective for the design of catalysts featuring multiple active sites and multifunctional components aimed at facilitating synergistic reactions.

Two-dimensional carbon materials, including graphene, are characterized by their single-layer structure composed of carbon atoms. They exhibit a high specific surface area, outstanding electrical conductivity, and remarkable thermal stability.^127^ The planar structure creates optimal conditions for the dispersion and adsorption of metal catalysts, simultaneously offering a substantial number of active sites. The conductivity of graphene significantly facilitates the swift movement of electrons, thereby improving the electrochemical performance of catalysts. An illustrative case is presented through the research conducted by Dao *et al.*,^128^ who developed an efficient two-site catalyst (LaCeO_*x*_@NGr/Ru_1_) for HER reactions in alkaline media. They achieved this by integrating oxygen vacancy-rich LaCeO_*x*_ with N-doped graphene-loaded monoatomic Ru. This study comprehensively elucidated the significance of graphene. Initially, the substantial specific surface area of graphene offered numerous anchoring sites for the incorporation of LaCeO_*x*_ and Ru single atoms, facilitating the uniform dispersion of active components and consequently enhancing the number of active sites within the catalyst (Fig. 8h). The remarkable electrical conductivity of graphene enhanced the electron transport process and minimized the electron transfer resistance, which played a vital role in optimizing the reaction kinetics of the HER reaction. Furthermore, the incorporation of graphene contributes to improved stability and durability of the catalyst, effectively preventing the agglomeration and detachment of active components throughout the reaction process. This study clearly illustrates the essential function of graphene in composites and offers important insights and references for the design and advancement of effective electrocatalysts. Innovative designs persist, reinforcing the potential of graphene substrates to improve catalytic performance, as demonstrated by the research of Hung *et al.*^129^ The potential of 2D carbon materials in enhancing HER performance was further demonstrated through the loading of Ru nanoparticles on graphene-based composites. This study successfully optimized catalyst activity and stability by adjusting the size and phase structure of Ru nanoparticles and integrating 3D composites of extended graphite and reduced graphene oxide (EG-rGO). The graphene substrate was found to provide numerous anchoring sites for Ru nanoparticles and to enhance electron transport due to its superior electrical conductivity, thereby significantly improving the efficiency of the HER reaction (Fig. 8i). Moreover, by accurately regulating the ratio of graphene to extended graphite and the annealing temperature, one can modify the microstructure and chemical composition of the composites to optimize HER performance. It is important to highlight that, alongside the aforementioned studies, there are additional related works that have explored the potential of 2D carbon materials in electrocatalysis. A study highlighted the significant contribution of 2D carbon materials in improving electrocatalytic performance, focusing on Co–Ru alloy nanoparticles loaded on nitrogen-doped two-dimensional carbon nanosheets (NCN). This study involved the preparation of Co–Ru-based catalysts (Co–Ru/NCN) using a one-step pyrolysis method. The findings indicated that the unique structure of 2D carbon nanosheets facilitated the availability of numerous active sites, while the cooperative interaction between Co and Ru markedly improved the catalytic efficiency. This study reaffirms the significance of 2D carbon materials in facilitating the dispersion of metal catalysts, augmenting the number of active sites, and improving electron transport efficiency.

**Fig. 9:**
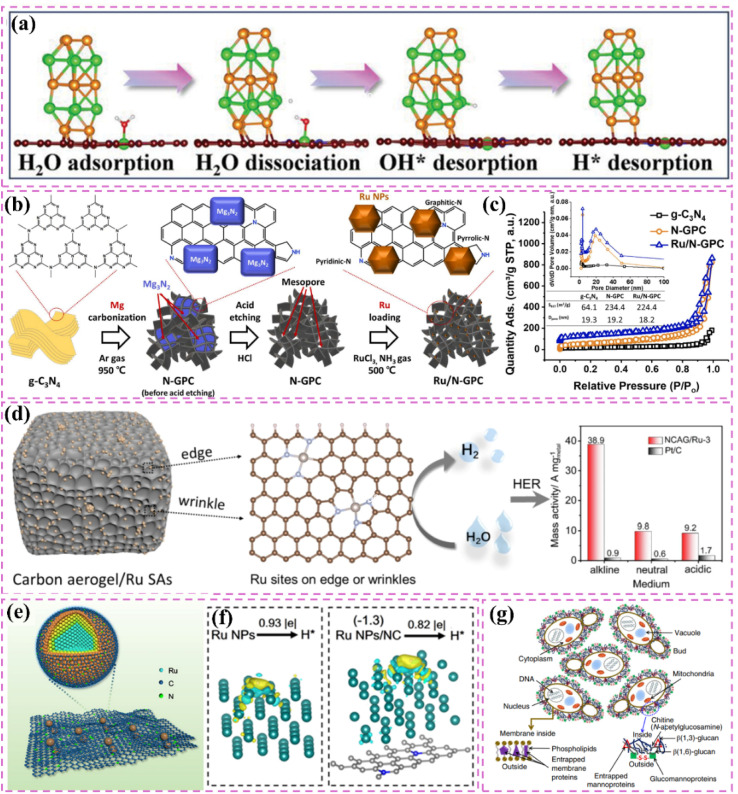
(a) Schematic illustration of alkaline HER mechanism for Ru_1_Co_1_ + RuCo model. Reproduced with permission from ref. 131. Copyright 2023, Wiley-VCH. (b) Schematic synthesis procedure for Ru/N-GPC. (c) N_2_ adsorption/desorption isotherms for various samples. Reproduced with permission from ref. 132. Copyright 2023, Elsevier. (d) Schematic diagram of the Ru sites in carbon aerogel as the pH-universal HER catalysts. Reproduced with permission from ref. 115. Copyright 2022, Elsevier. (e) Illustration of the formation of Ru NPs/NC. (f) Differential charge density diagrams, the isosurface is 0.015|*e*|, of H-adsorption state for Ru NPs and (−1.3) Ru NPs/NC models. Reproduced with permission from ref. 136. Copyright 2022, American Chemical Society. (g) A schematic yeast cell structure. Reproduced with permission from ref. 138. Copyright 2020, Springer Nature.

Three-dimensional carbon materials, including mesoporous carbon, exhibit a well-defined pore structure and a high specific surface area.^130^ Their adjustable pore size can significantly enhance the specific surface area of the catalyst and improve the accessibility of active sites. In the meantime, the pore structure of mesoporous carbon enhances the diffusion of reactants and products, thereby improving the mass transfer efficiency of the catalyst. The catalytic performance of 3D carbon materials can be enhanced by incorporating metal single atoms or nanoalloys onto their surface. For example, Wang *et al.*^131^ developed a RuCo bimetallic single-atom and nanoalloy coexisting catalyst (RuCo@Ru_SA_Co_SA_-NMC), which was supported on nitrogen-doped mesoporous carbon to attain outstanding HER performance in alkaline environments. This structure demonstrates that nitrogen-doped mesoporous carbon offers a wealth of active sites, enhances the adsorption and diffusion of water molecules, and facilitates faster reaction kinetics due to its distinctive pore architecture (Fig. 9a). Furthermore, the catalyst shows low overpotential and remarkable stability at elevated current density, highlighting the promise of 3D carbon materials in the development of highly efficient and stable industrial-grade HER catalysts. This accomplishment serves as a significant reference for advancing the creation of novel and efficient catalysts by leveraging the structural benefits of 3D carbon materials. Further investigation into the role of three-dimensional carbon materials in the electrocatalytic HER necessitates an analysis of the support effects and structural advantages of porous carbon materials. These materials function as efficient platforms for metal catalysts by providing numerous active sites. Their unique pore structures significantly enhance the mass transfer efficiency of reactants and improve the stability of the catalysts. Shin *et al.*^132^ synthesized a nitrogen-doped, highly graphitized porous carbon material (N-GPC) using a magnesium reduction method and employed it as a support for Ru nanoparticles (Ru/N-GPC) (Fig. 9b). This porous carbon material demonstrates a high specific surface area and an optimal pore size distribution, significantly improving the accessibility of active sites and enhancing the efficiency of the HER (Fig. 9c). The graphitized structure of N-GPC provides exceptional electronic conductivity, which reduces the overpotential of the hydrogenation reaction and accelerates reaction kinetics. The presence of pyridinic-N in the porous carbon material enhances the interaction between N-GPC and Ru nanoparticles, concurrently reducing the adsorption strength of hydrogen on the Ru nanoparticles. This dual action accelerates the Tafel step, reduces the overpotential of the hydrogenation reaction, and ultimately improves both the stability and activity of the catalyst.

He *et al.*^115^ conducted a related study that investigated the use of porous carbon materials in electrocatalytic hydrogen precipitation. They developed an atomically dispersed Ru-doped carbon aerogel (NCAG/Ru) and confirmed its efficient HER performance across a broad pH range (Fig. 9d). Carbon aerogels with atomically dispersed Ru and Ru nanoclusters were synthesized through the pyrolysis of biomass hydrogels. The presence of abundant microporous and nanofold structures in carbon aerogels effectively restricts the distribution of metal atoms and promotes the formation of isolated metal active sites. The active sites markedly enhanced the specific surface area of the catalyst and optimized the adsorption and activation processes of the reactants, leading to ultra-low overpotential and high HER activity. The three-dimensional porous structure of the carbon aerogel enhances the diffusion efficiency of reactants and products, thereby improving the mass transfer performance and stability of the catalyst. Theoretical calculations indicated that the RuN_*x*_ active sites located at the sawtooth edges and nanofolds of the carbon aerogels exhibit significant intrinsic activities, facilitating the adsorption and dissociation of water molecules as well as the adsorption and desorption of hydrogen. The study presents a novel approach and methodology for developing efficient and cost-effective pH-pervasive electrocatalytic hydrogen precipitation catalysts through the integration of a distinctive porous carbon material structure with atomically dispersed metal active sites.

Different dimensional carbon materials, with unique physicochemical properties, enhance the HER performance of carbon-supported Ru-based catalysts through a multi-level composite support system. However, while pursuing high performance, the industrial prospects of carbon supports must be critically assessed. For instance, zero-dimensional materials like fullerenes and carbon quantum dots are restricted by high costs and complex synthesis. Carbon nanotubes and graphene face challenges in cost-effectiveness and macro-structure control. In contrast, 3D porous carbons derived from biomass or polymers offer advantages like abundant raw materials, low cost, and easy processing, showing more potential for scaled-up applications. Thus, catalyst design should consider the availability, economics, and compatibility of support materials with industrial electrode preparation processes to pave the way for real-world applications of high-performance catalysts.

#### 3.1.2 Impact of surface chemistry on carbon supports

The surface chemical properties of carbon supports are pivotal in enhancing the performance of Ru-based HER by modifying the electronic microenvironment and reaction pathways at the active center through atomic-level interactions.^133^ The properties influence the active site and electronic structure of the catalyst while also playing a crucial role in the diffusion of reactants and products, as well as overall stability. Recent studies indicate that heteroatom doping, defect engineering, functional group modification, and hydrophilicity modulation can systematically influence metal–support electron transfer, interfacial bond strength, and mass-transfer kinetics. The mechanisms underlying these effects have been thoroughly elucidated through advanced characterization techniques. Fundamentally, both heteroatom doping and defect engineering aim to disrupt the electronic uniformity of the pristine carbon lattice. This results in localized sites with modified charge density and spin distribution, thus influencing the electronic structure of supported Ru species *via* metal–support interactions. This discussion will examine the impact of the surface chemistry of carbon supports on catalyst performance, supported by specific research examples.

Recently, various heteroatom-doped carbon materials have demonstrated notable advantages in electrocatalysis, becoming a key area of interest for enhancing catalyst performance. The introduction of different heteroatoms, including nitrogen, boron, sulfur, and phosphorus, into the carbon lattice has been demonstrated to notably alter the electronic structure, consequently enhancing both the activity and stability of the catalyst.^134^ Atoms exhibiting varying electronegativity and atomic radii relative to carbon can generate electron-rich or electron-deficient sites. Nitrogen doping generates Lewis basic sites that donate electrons to Ru, whereas boron doping produces electron-deficient sites that accept electrons. The electron transfer directly influences the d-orbital filling and d-band center of Ru, thereby fine-tuning the adsorption energy of intermediates such as H*. The underlying mechanism of the synergistic effect observed among various heteroatoms can be attributed to their distinct electronegativities and atomic radii. The variety present enables the creation of multiple active sites and electron transfer pathways in carbon materials, promoting efficient electron transfer and redistribution.^135^ This results in an optimized electronic environment of the metal active center, which enhances catalytic activity. The addition of different heteroatoms can improve the surface properties of carbon materials, such as hydrophilicity and pore structure, while also enhancing the interactions between the catalyst and reactants, along with the efficiency of mass transfer. Such improvement enhances the movement of reactants and the liberation of products, thereby boosting the speed and effectiveness of the catalytic reaction.

The investigation conducted by Jiang *et al.*^136^ explored the significant contribution of nitrogen-doped carbon to the improvement of catalyst performance (Fig. 9e). A novel Schottky catalyst was successfully designed by anchoring ultrafine Ru nanoparticles, which exhibit lattice compressive stress, onto nitrogen-doped carbon nanosheets. The study revealed that nitrogen doping can alter the electronic structure of carbon supports, facilitating the formation of Schottky junctions with Ru nanoparticles, thereby influencing the transfer and distribution of electrons. The alteration of the electronic structure enhanced the hydrogen adsorption capacity of the catalyst and markedly boosted its hydrogen generation performance in alkaline media (Fig. 9f). There exists a significant interaction between the nitrogen atoms in the nitrogen-doped carbon supports and the Ru nanoparticles. This interaction has the potential to modulate the electron density of the catalyst and enhance its adsorption capacity with the reaction intermediates, thereby improving the catalytic activity. Moreover, the incorporation of nitrogen significantly improves the stability and longevity of the catalysts, allowing them to sustain their outstanding performance throughout extended catalytic processes. This study offers insights and theoretical backing for the application of nitrogen-doped carbon materials in electrocatalysis, establishing nitrogen doping as an effective method for improving catalyst performance. The coordinated effect of multi-atom doping has been demonstrated to significantly improve the catalytic properties of carbon materials. The concurrent incorporation of various heteroatoms, including nitrogen and boron, into the carbon lattice can lead to enhanced modulation effects on the electronic structure. The combined effect not only optimizes the electron distribution of the carbon material but also improves the interaction between the metal active center and the reactants, thus boosting the activity and selectivity of the catalytic reaction. The research conducted by Wu *et al.*^137^ effectively developed catalysts that demonstrate outstanding HER activity in both acidic and alkaline environments through the incorporation of Ru into nitrogen and boron co-doped carbon nanomaterials. The simultaneous introduction of nitrogen and boron enhances the electronic structure of carbon materials while also promoting the effective dispersion of active metal particles, thereby improving both catalytic activity and stability. This study indicates that precise regulation of catalyst performance can be achieved by adjusting the structure and ratio of monomers, further validating the flexibility and universality of the synergistic doping strategy of multiple heteroatoms. This synergistic effect offers a crucial experimental foundation and theoretical backing for the development of novel, efficient electrocatalysts. A recent study by Tiwari *et al.*^138^ has further expanded the application prospects of multi-heteroatom doped carbon materials, particularly in sustainable energy. This study presents the successful preparation of a novel multi-heteroatom doped carbon (MHC) material utilizing waste yeast biomass as a precursor (Fig. 9g). The process involved carbonization and doping with heteroatoms, including nitrogen, phosphorus, and sulfur, for applications in HER and OER. In contrast to conventional preparation methods, the waste yeast cells utilized in this study exhibit a distinct three-dimensional structure and abundant biological components. These characteristics not only create an optimal environment for metal ion adsorption and homogeneous dispersion but also facilitate the formation of numerous active sites and pore structures during the carbonization process. The experimental results indicate that the doped carbon materials demonstrate superior electrocatalytic performance in an alkaline medium. This achievement offers a novel approach for the high-value utilization of waste biomass and paves the way for the development of low-cost, high-performance electrocatalysts. The catalysts preserved the three-dimensional structure of yeast cells and effectively anchored uniformly sized metal particles on their surface. The synergistic doping effect of multi-heteroatoms in these catalysts results in notable improvements in electronic structure modulation, active site exposure, and mass transfer efficiency. This multi-heteroatom doping strategy utilizing waste biomass enhances the preparation methods of carbon materials and offers innovative solutions to the dual challenges of energy and environmental issues.

Defect engineering involves the alteration of electronic properties and catalytic performance of carbon supports through the introduction of structural defects.^139^ Intrinsic defects within the carbon framework can be converted into centers for electronic modulation.^140^ Introducing oxygen defects in graphene can significantly enhance its adsorption capacity and catalytic activity towards metal catalysts.^141^ Oxygen defects were observed to influence the electronic structure of graphene, leading to a more heterogeneous surface charge distribution, which in turn enhances the interaction with metal catalysts. The synergistic effect of vacancy defects and carbon supports serves as an anchor site for metal active sites. Defects such as carbon vacancies produce low-coordinated carbon atoms with localized states that exhibit strong interactions with metal atoms. These sites frequently capture metal species, inhibiting aggregation. The modified local electronic environment at these defects can significantly induce charge redistribution in anchored Ru, thereby optimizing binding with reaction intermediates and reducing energy barriers for critical steps. This synergy provides the catalyst with enhanced activity and stability across various reaction conditions.

**Fig. 10:**
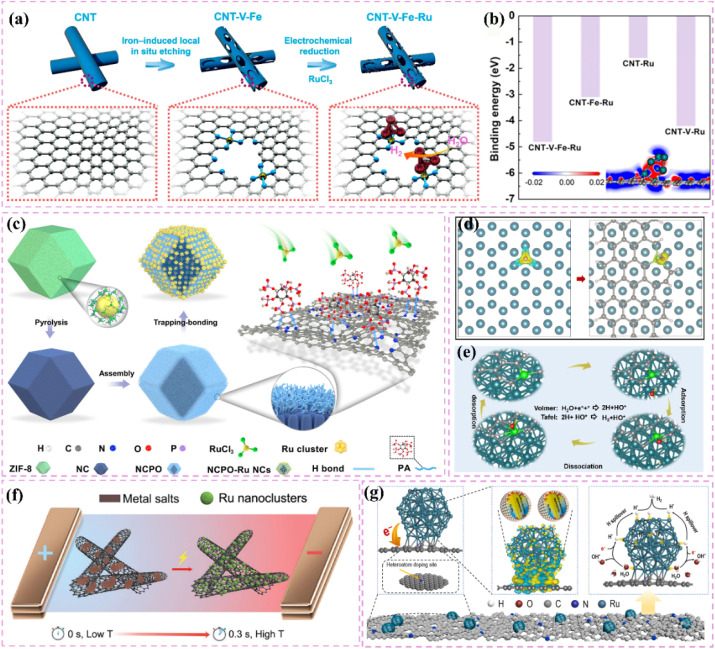
(a) Schematic of the preparation process of CNT–V–Fe as well as CNT–V–Fe–Ru and the HER catalytic process on Ru sites. (b) Binding energy between substrate and Ru clusters for CNT–Ru, CNT–Fe–Ru, and CNT–V–Fe–Ru and the charge density of the CNT–V–Fe–Ru model (the inset). Reproduced with permission from ref. 142. Copyright 2023, American Chemical Society. (c) Schematics of trapping–bonding strategy for the superassembly of surface-enriched Ru nanoclusters in water. Reproduced with permission from ref. 143. Copyright 2022, American Chemical Society. (d) OH differential charge density calculation results for Ru (002) and RuNi_def_/C models, in which color yellow indicates charge accumulation, and the color bright blue signifies charge depletion. (e) HER reaction process for RuNi_def_/C model under alkaline conditions. Reproduced with permission from ref. 101. Copyright 2024, Springer Nature. (f) Schematic illustration of the synthesis of Ru/O-CNT electrocatalysts. Reproduced with permission from ref. 144. Copyright 2024, Wiley-VCH. (g) Schematic illustration of mechanism of the enhanced HER activity on Ru/NDC. Reproduced with permission from ref. 145. Copyright 2023, Elsevier.

For example, a carbon nanotube (CNT–V–Fe) enriched with vacancy defects and monoatomic iron sites, prepared through an iron-induced functionalization strategy, has been documented (Fig. 10a).^142^ This structure serves as a support for Ru clusters and improves the HER performance of the catalyst in both acidic and basic environments. The study demonstrated that the combined influence of vacancy defects and iron monoatomic sites facilitated both the enhanced dispersion and stable growth of Ru clusters. Additionally, this interaction optimized the hydrogen adsorption strength of the Ru active sites by modifying the electronic structure of the carbon supports, leading to improved catalytic performance in HER (Fig. 10b). This study illustrates that the effectiveness of carbon supports in supporting metal catalysts can be markedly improved through the strategic design of defect structures. This work offers a fresh viewpoint and experimental foundation for utilizing vacancies and carbon supports in electrocatalysis. The work of Liang *et al.*^143^ further enriches the comprehension of how defect engineering can improve electrocatalytic performance (Fig. 10c). An innovative ‘capture-bonding’ strategy is presented to effectively enrich Ru nanoclusters on phytate-modified nitrogen-doped carbon frameworks (NCPO).

This approach effectively utilizes the nitrogen vacancy defects present in nitrogen-doped carbon frameworks, which exhibit high reducibility and strong interactions with the phosphate groups in phytic acid, thereby facilitating the effective trapping of Ru ions. The meticulous regulation of metal nanocluster distribution through defect engineering, coupled with the synergistic electronic interactions between the carbon supports and the metal active centers, results in the hydrogen precipitation activity of this NCPO-Ru NCs catalyst in alkaline electrolyte achieving 14.3 and 9.6 times that of the commercial Ru/C and Pt/C catalysts, respectively, while also demonstrating remarkable stability. This study offers fresh theoretical backing for the use of defect engineering and showcases its significant potential in renewable energy conversion *via* a practical solar-powered hydrogen production system, thereby broadening the scope of defect engineering in the development of effective electrocatalysts. Defect engineering strategies extend beyond vacancies and doping. They also encompass the enhancement of the synergistic effect between supports and active components through the construction of specialized structures. Yao *et al.*^101^ anchored nickel monoatoms on a defect-rich carbon support and incorporated ultra-small Ru nanoparticles to create a carbon bridge-connected monoatom-nanoparticle synergistic structure. The defective sites of the carbon supports serve as stable anchors for nickel monoatoms and facilitate efficient electron transfer among nickel, carbon, and Ru. This design overcomes the performance limitations of conventional noble metal catalysts, achieving low overpotential in alkaline conditions and maintaining long-term stability at elevated current densities. Experiments and theoretical calculations elucidate how the carbon bridge structure optimizes hydrogen adsorption strength and accelerates reaction kinetics, thereby highlighting the significant potential of defect engineering in improving the performance of non-precious metal catalysts (Fig. 10d and e). The catalysts exhibited significant activity and stability in both acidic and neutral electrolytes, highlighting the benefits of the defect engineering strategy in developing versatile pH-adaptive catalysts. This research offers important insights for the design of future high-efficiency, low-cost catalysts for water electrolysis.

**Fig. 11:**
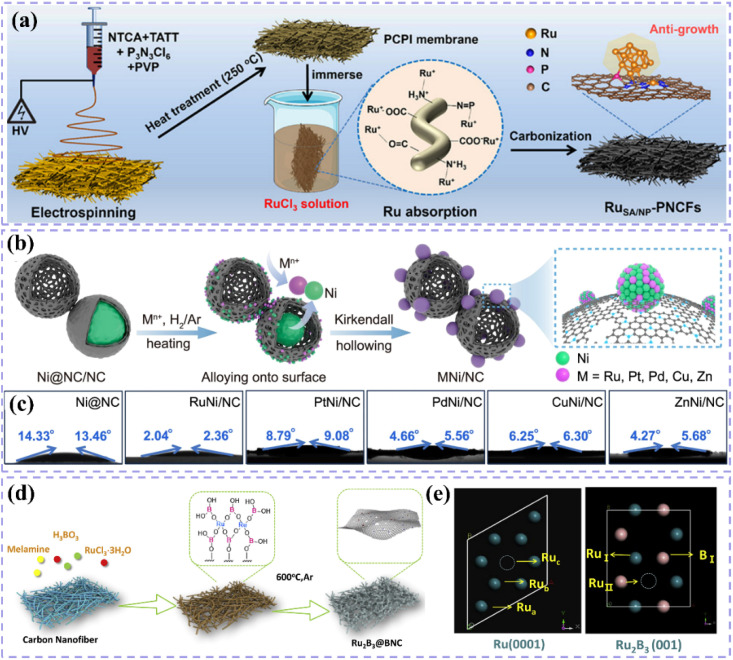
(a) Schematic illustration for preparing homogeneous Ru dispersed porous carbon fibers by *in situ* adsorbing method. Reproduced with permission from ref. 96. Copyright 2024, Elsevier. (b) Schematic illustration of the synthetic process of MNi/NC. (c) Optical images showing water contact angles of Ni@NC and MNi/NC series. Reproduced with permission from ref. 21. Copyright 2010, Springer Nature. (d) Synthetic illustration of the Ru_2_B_3_@BNC electrocatalyst. (e) The adsorption sites of H atoms on the modeled surfaces. Ru (0001) surface and Ru_2_B_3_ (001) surface. Reproduced with permission from ref. 150. Copyright 2020, Elsevier.

Functional groups, including carboxyl, hydroxyl, and epoxy groups, are typically found on the surfaces of carbon supports. These groups can form chemical bonds with metal catalysts, influencing the active sites and electronic structures of the catalysts. For instance, Wang *et al.*^144^ developed electron-deficient Ru nanoclusters (Ru/O-CNT) through a high-temperature impact method, utilizing oxygen-functionalized carbon nanotubes (O-CNT) as supports (Fig. 10f). This approach not only improved the electronic interactions between the metal and the support but also greatly enhanced the HER performance of the catalyst in alkaline conditions. An effective electronic metal–support interaction (EMSI) was achieved by anchoring Ru nanoclusters onto the surface of O-CNT and leveraging the oxygen functional groups on O-CNT to create chemical bonds with Ru. This interaction led to the transfer of electrons from Ru to O-CNT, maintaining Ru in an electron-deficient state, which subsequently optimized the adsorption strength of Ru with H* intermediates. The experiments demonstrated that the Ru/O-CNT catalyst displayed remarkable efficacy in the alkaline hydrogen evolution reaction. Moreover, the catalyst shows remarkable stability, highlighting its significant potential for real-world applications. The critical aspect of these studies is the modulation of the electronic structure of metal catalysts *via* functionalized carbon supports to enhance the efficiency of electrocatalytic reactions. Analogous studies have demonstrated that nitrogen functional groups effectively modulate the performance of metal catalysts. Ren *et al.*^145^ demonstrated that altering the nitrogen functional groups in nitrogen-doped carbon materials significantly influences the electronic asymmetry of the Ru nanoclusters anchored to them, thereby optimizing catalyst performance in alkaline HER. The researchers successfully altered the electronic distribution of Ru nanoclusters by modifying the types of nitrogen functional groups on nitrogen-doped carbon (NDC) supports, particularly by increasing the proportion of pyrrole-type nitrogen (pyrrolic-N). The experimental results indicated that Ru/NDC catalysts enriched with pyrrole-type nitrogen demonstrated superior HER activity in an alkaline medium. The optimization of electronic asymmetry accelerated water adsorption and dissociation while promoting hydrogen adsorption on the Ru surface, significantly enhancing the efficiency of the key step in the alkaline HER process (Fig. 10g). This study illustrates that the electronic structure of metal catalysts can be effectively adjusted by precisely modulating the nitrogen functional groups on carbon supports, offering a novel approach for the design of highly efficient electrocatalysts. Building on the aforementioned studies, Xu *et al.*^96^ investigated the influence of carbon support surface functional groups on the performance of metal catalysts. This study successfully constructed a structure featuring uniformly distributed Ru clusters alongside adjacent monoatomic Ru sites (Ru_SA_/NP-PNCFs) by introducing phosphorus into nitrogen-doped carbon fibers and employing phosphorus-doped polyimide nanofibers (PNCFs) as supports. The numerous functional groups (*e.g.*, –COOH, –NH_2_, –N=P) present on the surface of carbon supports exhibit a strong affinity for metal ions, effectively trapping and anchoring Ru^3+^ ions (Fig. 11a). This interaction leads to the uniform dispersion of ultrafine Ru clusters and monoatoms. The robust interaction enables Ru to preserve the stability of its electronic structure throughout prolonged catalysis. This study indicates that the functional groups on the surface of carbon supports can effectively regulate the active sites and electronic structures of catalysts *via* chemical bonding and electronic interactions with metal catalysts. Additionally, these functional groups significantly inhibit the aggregation and loss of metal clusters, leading to enhanced electrocatalytic performance and stability.

The hydrophilicity of the carbon support significantly influences the performance of the catalyst. Hydrophilic carbon supports enhance the diffusion of reactants and electrolytes, leading to improved activity and stability of the catalyst.^146^ For instance, carbon supports with high hydrophilicity can enhance electrolyte penetration, optimize the diffusion pathways of reactants, and minimize mass transfer resistance. Conversely, hydrophobic carbon supports could impede the diffusion of reactants and diminish the catalyst's performance.^147^ Modifying the hydrophilicity of the carbon supports allows for the optimization of the reaction environment and enhances the performance of the catalyst. Zhang *et al.*^21^ introduced a strategy termed “on-site disruption and near-site compensation” to enhance the catalyst-electrolyte interaction. This was achieved by synthesizing tip-like bimetallic RuNi nanocomposites (RuNi/NC) on superhydrophilic carbon nanocages (Fig. 11b). The elevated hydrophilicity and pronounced curvature of the carbon material contribute to a significant increase in active sites for the catalyst (Fig. 11c). Additionally, these characteristics improve electrolyte permeability and enhance reactant adsorption due to the material's distinct electronic properties and surface functional groups. The elevated specific surface area and porous architecture of carbon nanocages facilitate an optimal dispersion environment for metal nano-alloys, mitigating nanoparticle agglomeration and enhancing both the activity and stability of the catalysts. The elevated electrical conductivity of the carbon material facilitates swift electron transfer, a critical factor for electrocatalytic reactions. The experimental findings indicate that the RuNi/NC catalyst demonstrates significantly low overpotential and high stability in alkaline HER. The outstanding performances can be attributed not only to the tip effect of the catalyst and the synergistic effect of the metal components but also to the high hydrophilicity and superior physicochemical properties of the carbon material. Carbon materials serve not only as physical supports but also contribute through their distinctive chemical and electronic properties, which interact with metal nano-alloys to enhance the reaction kinetics and stability of the entire catalytic system. Nevertheless, it remains a problem to synthesize carbon-based catalysts that exhibit both high hydrophilicity and superior catalytic activity using a simple technique. A new solution was provided by Xie *et al.*^148^ in this context. They effectively synthesized a series of highly efficient Ru-based catalysts using the straightforward impregnation of bacterial cellulose (BC) with varying concentrations of Ru(bpy)_3_Cl_2_ solution, thereafter subjected to calcination under optimized conditions. The calcination process induced oxidation and molecular reorganization in the BC precursors, leading to the formation of superhydrophilic N-doped carbon nanofiber (CNF) matrices, which featured numerous oxygen-containing functional groups on their surfaces and exhibited a high water absorption capacity. The superhydrophilic carbon nanofibers not only strengthened the catalyst-electrolyte contact but also improved the diffusion efficiency of the reactants, hence augmenting the catalyst's performance. The influence of varying sizes of Ru nanoparticles on the HER activity of the catalyst was examined by DFT simulation, revealing that smaller Ru nanoparticles exhibit superior HER activity. The process of preparing the catalyst showed potential for scalability and held promise for industrial applications. This study illustrates the important role that hydrophilic carbon supports in enhancing catalytic performance. Enhancing the hydrophilicity of carbon supports efficiently improves the interface between the catalyst and the electrolyte, increasing the number of active sites and enhancing electron transfer efficiency, therefore augmenting the overall performance of the catalyst.

The surface chemistry of carbon supports has a significant effect on catalyst performance. Modulating surface functional groups, heteroatom doping, defect engineering, and hydrophilicity can significantly optimize the electronic structure and reaction environment of catalysts, thereby enhancing their catalytic performance. A comprehensive analysis of these properties will enhance our understanding of the structure–activity relationship of catalysts and offer critical insights for the design of more efficient carbon-supported Ru-based catalysts.

### 3.2 Strategies for enhancing carbon support performance

A comprehensive understanding of the catalytic mechanism of carbon-supported Ru-based catalysts indicates that optimizing the performance of carbon supports is essential for improving catalyst efficacy and overcoming existing application limitations. The regulation of electronic structure to optimize activity potential, the enhancement of mass transfer processes to accommodate high current density, and the improvement of stability for long-term reliable operation present numerous challenges and opportunities in each optimization direction. This section will detail specific strategies and research advancements in enhancing carbon support performance, offering significant insights and references for the development of high-performance carbon-supported Ru-based catalysts.

#### 3.2.1 Strategies for optimizing electronic structure

The pursuit of exceptional HER activity involves optimizing the interaction between catalyst active sites and reaction intermediates. The electronic structure of the active sites dictates this interaction. The primary strategy for improving intrinsic activity involves the meticulous design of the electronic structure of Ru sites and their carbon supports. All methods, despite their diversity, ultimately aim to establish an electronic environment that stabilizes transition states and lowers overall reaction barriers.

The optimization of the electronic structure of carbon supports is a fundamental approach to improving the HER performance of Ru-based catalysts. This strategy focuses on overcoming the limitations imposed by the volcano relationship between the d-band center and hydrogen adsorption strength by precisely modulating the charge transfer behaviors between the supports and the metal active sites. Recent studies have systematically altered the electronic microenvironment at the metal–support interface *via* heteroatom doping, defect engineering, and single-atom mediated strategies, thereby optimizing the energy barrier distribution of the reaction pathway.

Heteroatom doping serves as a fundamental method for optimizing electronic structure, effectively regulating the electron distribution of carbon supports and thereby improving catalyst performance. Nitrogen doping, through the introduction of nitrogen atoms, creates electron-rich regions within the carbon lattice, thereby altering the electronic properties of the carbon supports. The alteration in electronic structure can improve the interaction between the metal catalyst and the carbon support, optimize the electronic state of the metal catalyst, and consequently enhance catalytic activity. Qu *et al.*^149^ successfully prepared porous nitrogen-doped carbon supports (Ru/NC) loaded with ultrafine Ru species by utilizing the abundant nitrogen source and high porosity characteristics of nitrogen-doped COF. This structural design achieves high dispersion in Ru and further modulates its electronic structure *via* Ru–N bonding. The experimental results indicate that nitrogen doping markedly improves the interaction between Ru and carbon supports, thereby optimizing the electronic state of Ru. Nitrogen doping introduces active sites, including pyridine nitrogen, pyrrole nitrogen, and graphite nitrogen, which establish strong interactions with Ru species. This synergistic effect optimizes the electron transfer pathway and accelerates the kinetics of the HER. The integration of experimental data with theoretical calculations elucidates how nitrogen-doped carbon supports enhance catalytic performance through modulation of the electronic structure, improved metal dispersion, and increased reactant adsorption capacity. This result verifies the effectiveness of nitrogen doping in optimizing the electronic structure and serves as a significant reference for designing high-performance non-precious metal catalysts. Boron doping regulates the electronic properties of carbon supports through the introduction of boron atoms.

Boron atoms exhibit lower electronegativity compared to carbon atoms, and their doping results in the formation of an electron defect region within the carbon lattice, thereby altering the electron distribution of the carbon support. The electron defect region effectively adsorbs the metal catalyst, enhances the interaction between the metal catalyst and the carbon support, and optimizes the electronic structure of the catalyst. Qiao *et al.*^150^ demonstrated the advantages of boron doping in enhancing catalyst performance through the preparation of boron- and nitrogen-doped carbon nanofiber-loaded diruthenium tris-borides (Ru_2_B_3_@BNC) (Fig. 11d). The experimental results indicated that Ru_2_B_3_@BNC demonstrated superior HER activity across a broad pH range. DFT calculations indicate that the electron-deficient characteristics of boron atoms facilitate local electron redistribution, which increases the affinity for H* adsorption on Ru atoms. Boron doping introduces electronic defects that enhance metal–support interaction and optimize the free energy of hydrogen adsorption on the catalyst surface (Δ*G*_H*_) to near zero, significantly improving HER catalytic activity (Fig. 11e). This study illustrates that boron doping offers a novel approach for developing highly efficient and cost-effective non-precious metal-based catalysts through the modulation of electronic structure and adsorption characteristics.

**Fig. 12:**
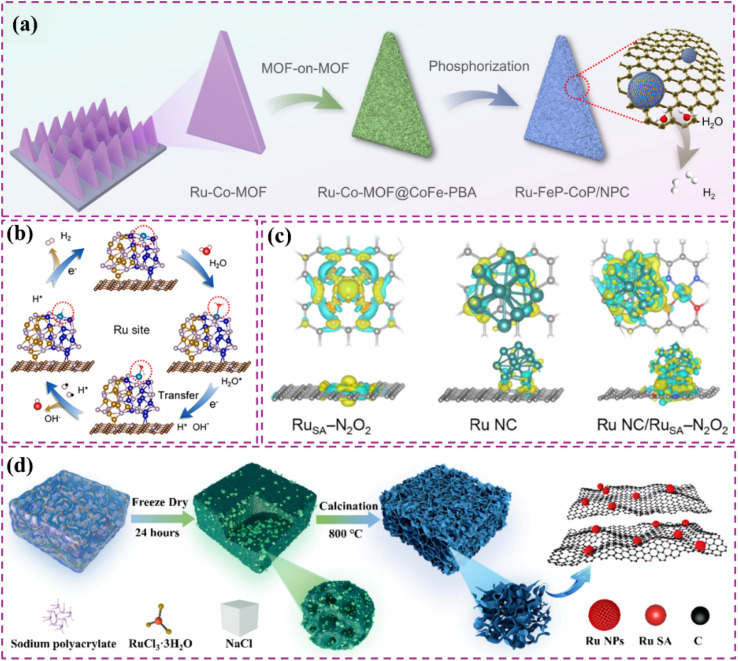
(a) Schematic sketch of the synthesis of Ru–FeP–CoP/NPC catalyst for HER. (b) Typical HER mechanism of Ru–FeP–CoP/NPC. Reproduced with permission from ref. 106. Copyright 2025, Wiley-VCH. (c) Charge density difference portrayal for Ru_SA_–N_2_O_2_, Ru NC, and Ru NC/Ru_SA_–N_2_O_2_ (yellow: electron accumulation, and cyan: electron depletion, and the isosurface is 0.02*e* Å^−3^). Reproduced with permission from ref. 117. Copyright 2025, Wiley-VCH. (d) Schematic illustration of the fabrication of Ru/C catalyst. Reproduced with permission from ref. 155. Copyright 2025, Elsevier.

Furthermore, phosphorus doping serves as an effective approach to modify the electronic structure of carbon supports. The electronegativity of phosphorus is comparable to that of carbon. However, phosphorus has a larger atomic radius. Doping can alter the lattice structure and electron distribution of carbon supports. Phosphorus doping introduces additional electron donor sites, modulates the electronic properties of carbon supports, and enhances the activity of metal catalysts. Long *et al.*^106^ synthesized FeP–CoP heterostructure-anchored single-atom Ru catalysts (Ru–FeP–CoP/NPC) through phosphorus doping, resulting in a notable enhancement in catalyst performance (Fig. 12a). The experimental results indicated that Ru–FeP–CoP/NPC demonstrated superior HER activity in alkaline environments. DFT calculations demonstrated that phosphorus doping optimized the electronic structure of the catalyst and weakened the strong adsorption of Ru–OH by modulating the local charge distribution, thereby accelerating the hydrolysis dissociation and hydrogen adsorption/desorption processes (Fig. 12b). The modulation of electronic structure and optimization of active sites allowed the phosphorus-doped catalysts to demonstrate enhanced catalytic efficiency and stability in the HER process. This study illustrates the efficacy of phosphorus doping in improving the performance of non-precious metal catalysts, offering a significant reference for the development of new efficient electrocatalysts. Heteroatom doping effectively regulates the electronic structure and chemical properties of carbon supports, enhances the interaction between metal catalysts and carbon supports, optimizes the electronic state and reaction environment of catalysts, and significantly improves catalyst performance.

Nanostructure engineering has the capability to effectively influence the electronic structure of carbon supports. Through meticulous manipulation of the nanostructures found in carbon materials, including nanoparticles, nanowires, and nanosheets, one can achieve a substantial enhancement in both the specific surface area and the quantity of active sites. Based on this, the design and preparation of single-atom catalysts (SACs) have further illustrated the significant impact of microenvironment modulation surrounding the active site on catalytic performance. For instance, Zhang *et al.*^151^ skillfully employed nanostructure engineering to enhance the spatial distribution and electronic characteristics of the active sites by anchoring Ru single atoms onto graphene-like frameworks and carbon-deficient tungsten carbide (WC_1−*x*_) substrates. The loading amount of Ru atoms is demonstrated to control the distance precisely between neighboring metal active sites, with moderate spacing significantly reducing the reaction energy barrier and thereby enhancing catalytic activity. The 0.76% Ru-loaded Ru-WC_1−*x*_ samples in this study demonstrated remarkable HER performance, achieving 100 hours of stable operation at a high current density of 1 A cm^−2^. This accomplishment underscores the significance of nanostructure engineering in enhancing the active sites of SACs while also serving as a valuable reference for the design of new electrocatalysts that exhibit high efficiency and stability.

The core strength of nanostructure engineering is its capacity to precisely modulate the local coordination environment of monatomic active centers, alongside spatial distribution and spacing control.^152^ Heteroatom doping (*e.g.*, N, P, S, B) and its configuration (pyridine N, graphite N, thioether S) in carbon supports, along with the introduction of specific defects, enable nanostructure engineering to precisely tailor the local coordination environments of monatomic active centers. This optimization enhances electron densities and adjusts the positions of d-band centers, facilitating fine-tuning of the adsorption energies of key reaction intermediates. Additionally, the strategy offers a foundation for the development of bimetallic or polymetallic single-atom sites that exhibit synergistic effects. Through meticulous design of the nanostructures, such as porous or layered configurations, it is possible to accurately anchor two or more distinct metal single atoms at designated sites, facilitating their spatial proximity and the potential formation of specific coordination arrangements. The interactions or co-activation of adjacent heterogeneous metal atoms can overcome the activity constraints associated with individual metal sites. The carbon support is essential to this process. This material offers a stable anchoring site for metal single atoms and modulates the metal–support interaction *via* its numerous defects and functional groups, which considerably influences the electronic structure and catalytic activity of the catalyst. Liu *et al.*^117^ demonstrated that Ru single atoms and nanoclusters can be effectively anchored by engineering carbon supports with targeted defects and functional groups, thereby optimizing the electronic environment surrounding the Ru nanoclusters at the RuSA–N_2_O_2_ sites (Fig. 12c). This carbon support's design enables the creation of dynamic hydrogen migration pathways and reduces excessive H* adsorption in the vicinity of the Ru clusters. Additionally, it promotes the generation and release of H_2_*via* consecutive H-binding channels, thereby significantly improving the catalyst's activity and stability. This achievement illustrates the significant role of carbon supports in the development of efficient two-site catalysts.

Nanostructure engineering influences the electronic structure at the atomic level and optimizes the meso/macro morphology of catalysts and mass transfer processes. The development of 3D hierarchical porous, hollow, or array structures significantly enhances the accessibility of active sites, thereby promoting the diffusion of reactants into these sites and improving the mass transfer efficiency of products exiting them.^154^ Such improvement is essential for reactions requiring operation at elevated current densities, such as industrial-grade electrolytic water production. Recent studies have demonstrated substantial enhancements in catalytic performance through the rational design of catalyst nanostructures. The 3D ordered macroporous Ru–CoP@NC electrocatalyst (3DOM Ru–CoP@NC) developed by Zhang *et al.*^153^ effectively illustrates the benefits of nanostructure engineering. This study presents Ru–CoP@NC electrocatalysts featuring 3D hierarchically ordered macroporous structures, which were synthesized from ordered macroporous-microporous metal–organic frameworks (MOFs) using template and dual-solvent methods. The distinctive structural design allowed the catalyst to achieve a high specific surface area and numerous active sites while promoting electrolyte penetration and rapid electron transport. The improved performance is primarily due to the three-dimensional, highly interconnected mesh nanospaces and the synergistic interaction between the N-doped carbon framework and the Ru and CoP nanoparticles, which illustrates the importance of nanostructural engineering in optimizing catalyst performance.

Nanostructure engineering is a crucial approach to improving the stability of SACs, effectively preventing migratory agglomeration and leaching deactivation of single atoms by enhancing metal–support interactions, utilizing protective shell layer structures, or constructing robust anchoring sites. The significant impact of nanostructure engineering on catalyst performance was supported by the study conducted by Wang *et al.*^155^ an electrocatalyst (Ru/C-800) was synthesized with exceptional performance using a self-templating strategy that simultaneously anchored Ru single atoms and Ru nanoparticles on graded porous carbon (Fig. 12d).

This catalyst demonstrates superior HER catalytic activity and overall water decomposition in acidic and alkaline solutions, necessitates low overpotentials, and presents a minimal Tafel slope. The exceptional performance can be primarily attributed to the synergistic effect arising from the co-existence of graded porous carbon with Ru single atoms and Ru nanoparticles. This study confirms the effectiveness of nanostructure engineering in enhancing catalyst performance and proposes a straightforward self-templated synthesis strategy, offering a novel approach for designing catalysts with high catalytic activity across a broad pH range. In summary, nanostructure engineering effectively optimizes the electronic structure of carbon supports. The performance of catalysts is significantly enhanced through the modulation of the coordination environment, the construction of synergistic effect sites, the optimization of morphology, and the improvement of stability across various dimensions. The ongoing advancement of nanotechnology will significantly enhance the role of nanostructure engineering in the creation of novel and efficient catalysts.

#### 3.2.2 Strategy enhanced by transmission

**Fig. 13:**
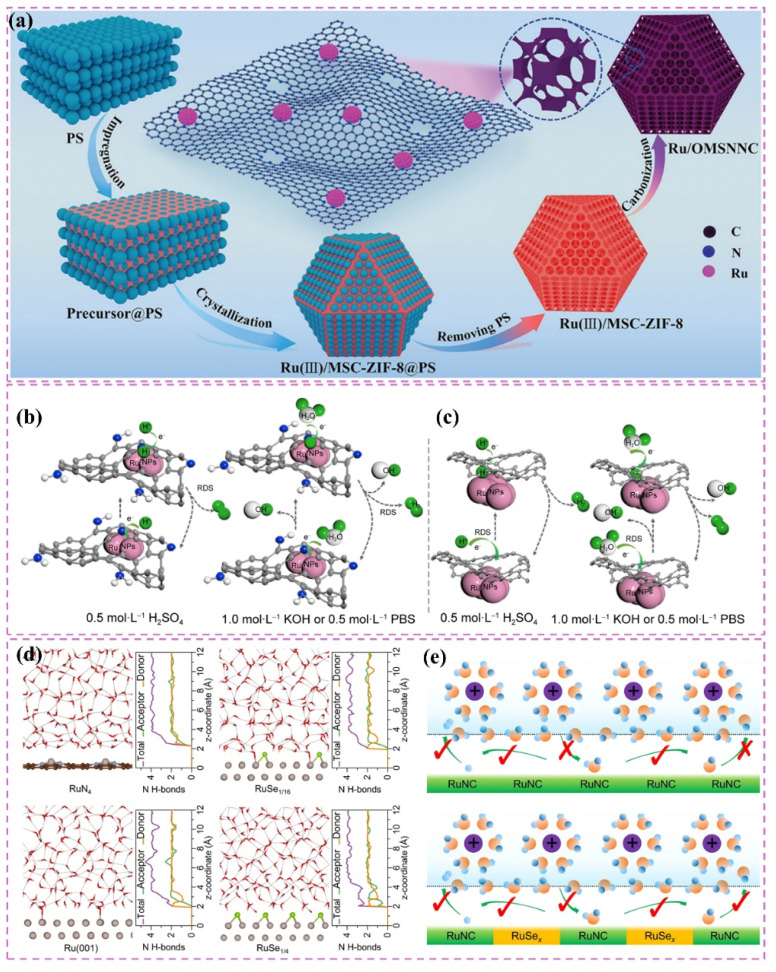
(a) Schematic representation for the preparation of the Ru/OMSNNC micro/nanoreactors. Reproduced with permission from ref. 36. Copyright 2021, Wiley–VCH. Reaction mechanism of (b) Ru/S-HMCs, and (c) Ru@HMCs in different electrolytes (blue, white, gray, and green spheres representing S, O, C, and H atoms, respectively. RDS: rate-determining step). Reproduced with permission from ref. 157. Copyright 2023, Springer Nature. (d) The representative snapshots of the structure of interfacial water molecules and the corresponding average distribution of hydrogen bond number along the surface. (e) Proposed mechanisms for neutral HER over RuNC (left) and RuSe_*x*_–RuNC (right). Reproduced with permission from ref. 51. Copyright 2022, Springer Nature.

Improving the mass transfer process is crucial for enhancing catalyst performance at elevated current densities. Constructing multistage pore structures effectively optimizes the mass transfer pathway.^156^ The multi-stage pore structure integrates the benefits of micro-, meso-, and macro-pores. Micro-pores offer a high specific surface area and numerous active sites, while meso- and macro-pores enhance the rapid diffusion of reactants and products. Wu *et al.*^36^ reported an electrocatalyst synthesized through the pyrolysis of ordered macroporous ZIF-8 single crystals containing Ru(iii) ions to achieve this goal. The catalyst features an ordered macroporous superstructure composed of nitrogen-doped nanoporous carbon, with ultrafine Ru nanoclusters anchored onto it. The Ru(iii)/MSC-ZIF-8 precursor exhibits a highly ordered macroporous structure. The evaporation of Zn nodes during pyrolysis results in nitrogen-rich defective sites, facilitating the stable anchoring of Ru nanoclusters (Fig. 13a). This study demonstrated that a hierarchical pore structure comprising macro-, meso-, and micropores can markedly improve the mass transfer efficiency of catalysts in comparison to traditional microporous carbon supports. Macropores serve as reservoirs for the electrolyte and facilitate the rapid transfer of reactants and products during reactions. Mesopores improve the accessibility of active sites, enabling fuller participation in the reaction. Micropores offer a high specific surface area and a significant number of active sites, thereby enhancing the overall activity of the catalyst. The results demonstrate that an optimized pore structure design enhances mass transfer and significantly improves catalyst performance at elevated current densities, offering a crucial reference for the advancement of electrocatalytic micro/nano reactors in future energy applications.

The mesoporous carbon support enhances the specific surface area and the accessibility of active sites in the catalyst, attributable to its uniform pore structure and elevated specific surface area. The pore structure enhances the diffusion of reactants and products while minimizing mass transfer resistance. The adjustable pore size of mesoporous carbon supports optimizes the reaction environment for catalysts, thereby enhancing their catalytic performance. Ma *et al.*^157^ elucidated the mechanism by which varying anchoring positions of Ru in mesoporous carbon (outer surface, cavity, and pore channel) influence catalyst performance. Catalysts positioned on the outer surface of mesoporous carbon demonstrated superior specific area activity, which was attributed to the complete exposure of active sites. In contrast, catalysts located within the pores exhibited enhanced mass activity and economic efficiency, resulting from their high dispersion and atom utilization (Fig. 13b and c). This result serves as a significant reference for designing new high-efficiency catalysts. By optimizing pore size and active site distribution, efficient electrocatalytic reactions can occur across a broader pH range, thereby reinforcing the crucial role of mesoporous carbon supports in improving catalyst performance.

Surface modification and functionalization, achieved through the introduction of specific functional groups or molecules to the surface of carbon supports, can substantially enhance their surface chemical properties and catalytic performance.^158^ Modifying the surface of the carbon support with hydrophilic functional groups enhances its hydrophilicity. This procedure facilitates the infiltration of electrolytes and the diffusion of reactants, thereby decreasing mass transfer resistance. Conversely, the introduction of hydrophobic functional groups can modify the surface hydrophobicity of the catalyst.^159^ This optimizes the adsorption and desorption processes of reactants under specific reaction conditions, thereby enhancing the catalyst's performance. Sun *et al.*^51^ demonstrated the successful modulation of interfacial water structure in a neutral medium through the introduction of nitrogen-coordinated Ru monoatom (RuNC) and Ru selenide (RuSe_*x*_) clusters on a carbon support surface, resulting in a significant enhancement of HER performance. This study demonstrates that modulating the hydrogen bonding network of interfacial water molecules transforms the interfacial water layer from an ordered to a disordered state (Fig. 13d). This alteration enhances the transport of water molecules and hydroxide ions at the electrode/electrolyte interface, thereby significantly improving the catalyst's activity and stability (Fig. 13e). This example demonstrates that the strategic design and incorporation of surface functional groups are essential for optimizing catalyst performance, particularly in modulating hydrophilicity. The modulation of hydrophilicity optimizes electrolyte penetration and reactant diffusion while improving the interaction between the catalyst and the reaction medium, thereby enhancing catalytic reaction efficiency. The rational design of multilevel pore structures, selection of appropriate mesoporous carbon supports, and surface modification and functionalization can significantly optimize the mass transfer performance of catalysts, thereby overcoming the limitations of high current density and improving overall performance.

The creation of a multi-level porous structure has enhanced the mass transfer efficiency of electrodes. However, its performance at high current densities is critically constrained by bubble management challenges. Inefficient bubble discharge can obstruct the pores, negate the benefits of mass transfer, and significantly reduce performance. This significant factor is frequently neglected in contemporary research. Future electrode designs must integrate hydrophilic and hydrophobic regulation with porous structures to facilitate the synergistic transport of reactants, ions, and product bubbles. Most multi-level structures exhibiting superior mass transfer performance currently depend on intricate template-based synthesis methods. The complex processes and template removal procedures elevate production costs and present environmental challenges. From an industrial standpoint, the development of template-free or recyclable-template green synthesis methods is essential. Furthermore, a notable disparity exists between performance evaluations conducted at the laboratory scale and the actual mass transfer conditions in industrial-scale electrolyzers, particularly those featuring large 3D electrodes. Systematic validation studies under simulated industrial operating conditions are urgently required to enable the transition of porous electrodes from ideal models to practical engineering applications.

#### 3.2.3 Strategy for enhancing stability

**Fig. 14:**
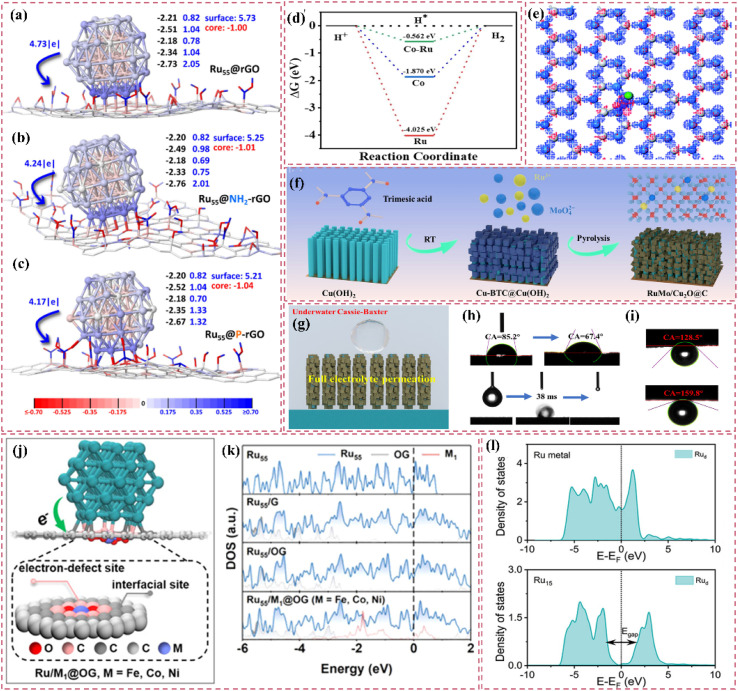
d-Band center and MPA charge analysis of Ru_55_@rGO (a), Ru_55_@NH_2_-rGO (b), and Ru_55_@P-rGO (c). Reproduced with permission from ref. 160. Copyright 2025, American Chemical Society. (d) DFT-calculated free-energy change for HER of Co–Ru heterostructures, Ru, and Co. Reproduced with permission from ref. 161. Copyright 2022, Elsevier. (e) The electron density difference of M4 with the adsorbed H atom. Reproduced with permission from ref. 162. Copyright 2021, Royal Society of Chemistry. (f) The schematic process for synthesizing RuMo/Cu_2_O@C catalyst. (g) The underwater Cassie–Baxter model, and (h) the captured rapid water diffusion process on the surface of RuMo/Cu_2_O@C, and (i) the underwater air contact angle on the CF and RuMo/Cu_2_O@C, respectively. Reproduced with permission from ref. 163. Copyright 2025, Wiley-VCH. (j) Atomic structures of Ru_55_ supported on O-doped graphene with dispersed metal atoms obtained by DFT calculations. (k) Density of states (DOS) for Ru_55_/M_1_@OG. The occupied states were shadowed. The dashed line indicates the Fermi level for each system. Reproduced with permission from ref. 167. Copyright 2021, Wiley-VCH. (l) Partial density of states of the Ru d-orbital of Ru metal and Ru nanoparticle (15 elements) on Fe_3_O_4_. *E*_F_ indicates the Fermi level. Reproduced with permission from ref. 168. Copyright 2023, Wiley-VCH.

Improving the stability of the catalyst is key to preserving its outstanding performance during prolonged reactions. The stability of the carbon support is crucial for the longevity of the catalyst. Carbon supports with high stability can significantly inhibit structural alterations or corrosion throughout the reaction process, thereby extending the catalyst's operational lifespan. The exceptional stability and mechanical strength of graphene enable it to effectively support Ru nanoparticles, preventing agglomeration even under challenging reaction conditions. Moreover, the surface functional groups and defects present on the carbon supports can establish robust interactions with the catalyst, thereby further improving its stability. Through the thoughtful design of the structure and surface characteristics of carbon supports, one can attain effective stabilization of catalyst nanostructures. Mallón *et al.*^160^ synthesized and characterized reduced graphene oxide-loaded Ru nanoparticles doped with nitrogen (NH_2_-rGO) and phosphorus (P-rGO) through a combination of experimental and theoretical methods to assess their electrocatalytic activity for HER in acidic environments. Doping was found to significantly enhance the stability of the catalysts, particularly the phosphorus-doped material, Ru@P-rGO, which demonstrated exceptional electrocatalytic performance with minimal degradation during constant current density experiments lasting up to 12 hours. The enhancement of stability is due to the electronic effects and structural defects caused by the dopant atoms. These factors improve the interaction between Ru nanoparticles and support, effectively preventing the agglomeration and oxidation of the nanoparticles, thereby ensuring the catalyst's high efficiency and stability over extended periods. The impact of doping on the electronic structure of Ru nanoparticles was thoroughly examined through DFT calculations (Fig. 14a–c). The study highlighted how doping-induced alterations in the d-band center and charge transfer modulate catalytic activity, offering theoretical guidance for optimizing catalyst support design through doping. This study confirms the significant impact of carbon support stability on catalyst performance and provides an informative guide to the advancement of new efficient and stable HER electrocatalysts.

Enhancing catalyst stability hinges on the modulation of metal–support interactions. Robust interactions between metal and support can significantly prevent the dissolution and agglomeration of catalysts, thereby improving their stability throughout the reaction process. Zhao *et al.*^161^ presented a new catalyst structure comprising Ru-modified cobalt nanoparticles supported on nitrogen-doped carbon flakes (Ru/Co@NC) for efficient HER across the entire pH spectrum. Nitrogen-doped carbon flakes served as supports for cobalt nanoparticles and established strong interactions with them, effectively preventing dissolution and agglomeration during the catalysis process, thereby enhancing the stability of the catalyst. The electrochemical test results indicated that the current loss of Ru/Co@NC remained negligible following an extended HER test, demonstrating its stability across a broad pH range. Theoretical calculations indicated that Ru/Co@NC exhibits a lower H_2_O adsorption energy and a Δ*G*_H*_ value closer to zero, facilitating more efficient water dissociation and H* adsorption/desorption during the HER process (Fig. 14d). This research presents a novel approach for developing high-efficiency, low-cost HER catalysts and elucidates the significant role of metal–support interactions in electrocatalysis. In a related study, Li *et al.*^162^ showed that the stability and activity of catalysts can be notably improved by adjusting the interactions between the metal and the support. The team developed defect-rich Ru/g-C_3_N_4_ nanosheet catalysts using a straightforward pyrolysis method, demonstrating outstanding HER performance in both acidic and alkaline electrolytes. There were effective coordination interactions between Ru and g-C_3_N_4_, which not only improved the electronic structure but also greatly increased the stability and activity of the catalysts. Theoretical calculations indicated that the effective orbital hybridization between Ru and N enhanced electron transfer and the adsorption of reaction intermediates, thereby accelerating the kinetics of the HER (Fig. 14e). This study offers a significant theoretical and experimental foundation for advancing high-performance Ru-based catalysts through the modulation of metal–support interactions.

Furthermore, the design of nanostructures plays a crucial role in improving the stability of catalysts. The development of core–shell or hollow structures serves to isolate the active center effectively, preventing direct interaction with the reaction medium and thereby minimizing corrosion and dissolution of the active center. For example, Yang *et al.*^163^ systematically synthesized core–shell nano-arrays to modulate interfacial water activity and hydrogen bubble behavior, resulting in a significant enhancement of the stability and catalytic activity of the catalysts at high current densities. This study demonstrates that the core–shell nanoarrays, developed from MOF-derived oxide materials, successfully attained a uniform distribution of active centers with minimal Ru content (Fig. 14f). Additionally, they efficiently released numerous small hydrogen bubbles through vertical channels, preventing the blockage of active sites and surface damage. The experimental results indicated that the core–shell catalyst maintained stable operation in 1 M KOH seawater at an ultra-high current density of 2 A cm^−2^ for over 800 hours, showcasing remarkable long-term stability. Additionally, DFT calculations revealed that the incorporation of highly dispersed Ru and Mo species adjusted the Δ*G*_H*_ of the catalyst to nearly zero, thereby enhancing its catalytic performance. In the meantime, the distinctive core–shell architecture of the catalyst provided it with remarkable superhydrophilic and underwater supergas-phobic characteristics, which effectively minimized the attachment of hydrogen bubbles to the catalyst surface and promoted the swift release of gas bubbles, thereby considerably improving the stability and longevity of the catalyst in seawater electrolysis (Fig. 14g–i). This outcome unequivocally validates the practicality and efficacy of enhancing catalyst stability *via* nanostructure design, offering a significant reference for advancing the development of efficient and stable catalysts for water electrolysis.

Through the careful selection of stable carbon supports, the enhancement of metal–support interactions, the execution of interfacial engineering, and the design of robust nanostructures, it is possible to effectively suppress the dissolution and agglomeration of catalysts. This approach significantly improves their stability and durability, ensuring optimal performance in long-term reactions. The strategies outlined here offer a significant theoretical foundation and practical insights for advancing high-performance, long-lasting carbon-supported Ru-based catalysts.

#### 3.3 Mechanisms of interactions between supports and catalysts

Research on carbon-supported Ru-based catalysts necessitates a thorough examination of the interaction mechanisms between supports and catalysts. The interactions affect the electronic structure and active-site distribution of the catalyst, altering its interaction with reactants and significantly impacting catalytic performance. Comprehending these intricate mechanisms elucidates the operational principles of catalysts at the microscopic scale. This establishes a robust theoretical framework and delineates a clear research trajectory for the design of novel, efficient, and stable carbon-supported Ru-based catalysts. We will examine interfacial electron-transfer effects and nanoscale structural stability mechanisms to elucidate their influence on catalyst performance and offer vital clues for catalyst design and optimization.

#### 3.3.1 Effect of interfacial electron transfer

In the study of carbon-supported catalysts, the effects of interfacial electron transfer are fundamental to understanding catalytic performance. A common example is the strong interaction between metal and support (SMSI).^164^ The process of SMSI entails the transfer of electrons between the metal and the support, driven by their robust interaction. This interaction modifies the electronic structure of the metal, leading to improved catalytic performance. For instance, the robust interaction between Pt and cerium dioxide alters the electron cloud density on the surface of Pt nanoparticles, which inhibits sintering and enhances catalytic activity.^165^ The electron-transfer effect becomes increasingly pronounced under high-temperature or reactive conditions, leading to a notable enhancement in catalyst stability.

The interaction between electronic supports and metals, known as electronic metal–support interaction (EMSI), plays a crucial role in the phenomenon of SMSI, emphasizing the charge redistribution that occurs between these entities.^166^ In metal catalysts loaded with graphene, the π-electron cloud of graphene interacts with the d-orbitals of the metal, resulting in a charge transfer that alters the position of the d-band center of the metal and influences the strength of reactant adsorption. The interaction in HER serves to lower the reaction energy barrier, thereby enhancing catalytic efficiency. Su *et al.*^167^ proposed the dispersion of individual metal atoms on oxygen-doped graphene to modulate the electrocatalytic behavior of loaded metal nanoparticles. The ideal atomic metal species were computationally screened and subsequently validated through experiments involving Ru nanoparticles on oxygen-doped graphene, which were adorned with individual metal atoms including Fe, Co, and Ni. The findings indicated that this hybrid catalyst demonstrated outstanding performance in HER. Subsequent analyses indicated that the transfer of charge from isolated metal atoms (such as Fe, Co, and Ni) to oxygen-doped graphene transformed the typically inert carbon surface into a more reactive one, thereby improving its interaction with metal nanoparticles (Fig. 14j). This interaction facilitates charge transfer between the metal nanoparticles and the support while also resulting in a shift in the position of the center of the d-band of the metal nanoparticles, subsequently influencing the adsorption strength of the reactants (Fig. 14k). The optimized electronic structure in the HER process led to a more appropriate adsorption strength of H* on the catalyst, thereby reducing the reaction energy barrier and enhancing catalytic efficiency. This study introduces a novel approach to improve the electrocatalytic activity and stability of metal nanoparticles through precise regulation of their electronic structure. It also serves as a significant reference for the design and development of high-performance non-precious metal electrocatalysts.

Moreover, SMSI influences the adsorption and activation mechanisms of reactant molecules on the catalyst surface. This effect increases the catalyst's ability to adsorb reactants, lowers the activation energy required for the reaction, and boosts catalytic efficiency. Catalysts exhibiting the SMSI effect demonstrate a significant ability to modulate hydrogen adsorption and reduction processes, thereby enhancing reaction selectivity and yield. This interfacial electron transfer phenomenon offers a significant theoretical foundation and practical insights for enhancing the electronic structure and boosting the catalytic efficiency of catalysts. Li *et al.*^168^ formulated an adaptive SMSI-based approach to deposit Ru nanoparticles onto iron tetraoxide (Fe_3_O_4_) and carbon support, resulting in Ru–Fe_3_O_4_/C for alkaline HER. The interaction of Ru nanoparticles with Fe_3_O_4_ and carbon support resulted in electron redistribution, thereby modifying the adsorption behavior of the catalyst. The Ru–O bonds, formed *via* orbital hybridization, modified the charge state of the surface Ru sites, facilitating increased electron flow to H*, thereby enhancing adsorption. This interaction enhanced the properties of H and antibonding, promoted the reorganization of H species, and accelerated hydrogen molecule formation (Fig. 14l). This study experimentally confirms the substantial impact of SMSI on catalytic performance and elucidates the underlying mechanism through comprehensive spectroscopic evidence and theoretical calculations. This study establishes a significant theoretical foundation for the design and development of innovative and efficient catalysts, highlighting the substantial potential of enhancing catalytic performance through the modulation of SMSI.

The SMSI presents both advantages and challenges. Although it has the potential to stabilize metal nanoparticles and enhance their electronic structure, an overabundance of SMSI might lead to the support partially or entirely encasing active sites, thereby diminishing their accessibility and resulting in catalyst deactivation. Therefore, attaining the ideal equilibrium between stability and activity through meticulous regulation of SMSI during synthesis presents a nuanced but essential challenge in the realm of catalyst design. Interpreting performance enhancements from metal–support interactions necessitates a clear distinction between interfacial electronic effects and hydrogen spillover effects. The former enhances the adsorption energy of reaction intermediates on active sites by modifying the metal's electronic state, exemplifying electronic-level regulation. The latter entails secondary hydrogen atom transfer and reactions occurring at various sites. Performance gains often attributed to spillover may, in fact, stem from significant interfacial electronic effects. In the design of experiments and arguments, it is essential to prioritize and verify universal electronic effects with rigor. In summary, SMSI plays a crucial role in the functionality of carbon-supported catalysts. The modification of the electronic structure of metal catalysts leads to improved catalytic performance and stability. The systematic regulation of the SMSI effect can offer significant insights for advancing high-performance catalysts.

#### 3.3.2 Mechanisms for stabilizing nanostructures

In the study and utilization of carbon-supported Ru-based catalysts, researchers encounter the challenge of maintaining structural stability during prolonged operation and under severe conditions, alongside the pursuit of high activity. Catalysts frequently experience phenomena such as Ostwald ripening and migratory agglomeration within intricate electrochemical settings, resulting in a reduction of active sites and subsequent performance decline.^169^ Carbon supports effectively mitigate the unstable behavior of Ru nanoparticles through a dual mechanism involving physical domain limitation and chemical bonding, thereby ensuring the structural stability of the catalyst. This paper will examine the nanostructural stabilization mechanism, emphasizing the synergistic effects of the domain-limiting effect and bonding engineering in enhancing catalyst stability, thereby providing invaluable information for catalyst design and optimization.

**Fig. 15:**
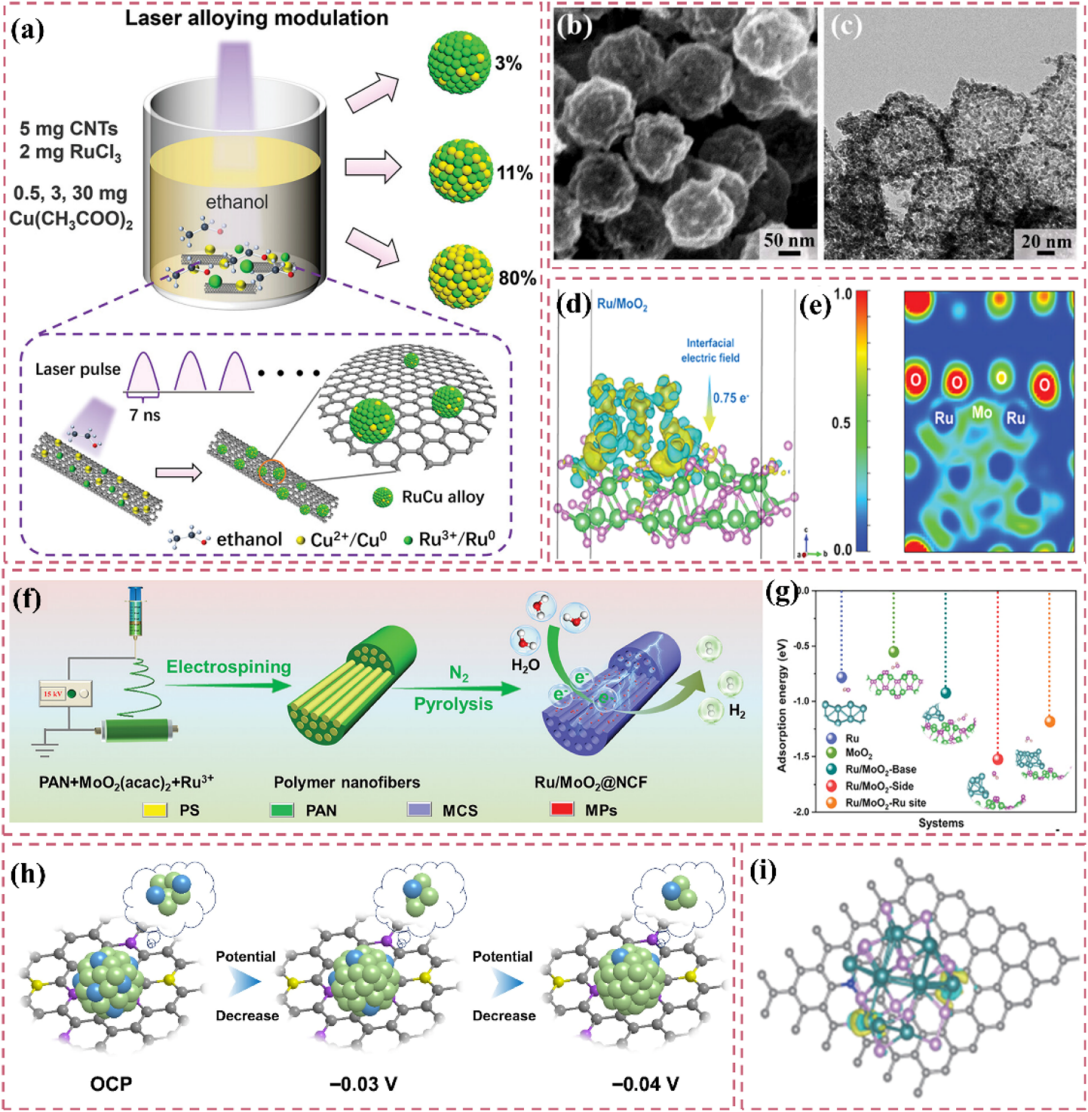
(a) Schematic of synthesis of RuCu_*x*_ (*x*: atom percentages of 3%, 11%, 80%) alloys by changing metal ratios of the RuCl_3_ and Cu(CH_3_COO)_2_ ion precursor. Reproduced with permission from ref. 170. Copyright 2025, Wiley-VCH. (b) SEM and (c) TEM images and enlargement image. Reproduced with permission from ref. 171. Copyright 2024, Springer Nature. (d) Charge density difference at the interface of Ru and MoO_2_, the blue and yellow regions reflect electron-deficient state and electron-rich area, respectively. (e) Electron localization function evaluations of Ru/MoO_2_ heterostructure. (f) Schematic illustration of the fabrication process. (g) H_2_O adsorption energy and sites of Ru, MoO_2_, and Ru/MoO_2_. Reproduced with permission from ref. 109. Copyright 2024, Wiley-VCH. (h) The scheme of catalytically active RuCo SAA is proposed based on *operando* XAS analysis. Reproduced with permission from ref. 173. Copyright 2025, Springer Nature. (i) The charge difference of RuP@NPC. Reproduced with permission from ref. 174. Copyright 2021, Springer Nature.

The domain-limited effect markedly improves catalyst stability by obstructing particle migration pathways *via* nanoscale spatial barriers. For instance, utilizing the presence of defects and functional groups on the surface of CNTs, metal nanoparticles establish a robust anchoring effect on their surface.^170^ The combination of this anchoring effect with laser-induced ultrafast heating and cooling processes resulted in the synthesized Ru–M alloy nanoparticles being tightly embedded in specific regions of the carbon nanotubes, thereby creating a physically spatially confined domain (Fig. 15a). The nanoscale spatial segregation successfully prevents the migration and collision of metal particles during high-temperature reactions, thereby greatly enhancing the thermal and chemical stability of the catalyst. Furthermore, the cage-like architecture and confined domain of carbon nanotubes can significantly improve the interaction between the metal and the carbon support, resulting in robust metal-substrate bonding. In Ru-based catalysts, hollow carbon sphere supports demonstrate notable stabilization benefits. Their internal cavities function as nanoreactors, effectively restricting the volume expansion and lattice distortion of the active components throughout the reduction process. An example of a successful study involves the synthesis of nitrogen-doped carbon (NC)-coated porous Ru/RuO_2_ heterojunction hollow nanospheres (Ru/RuO_2_/NC) achieved through a straightforward polymeric assembly and subsequent calcination processing strategy.^171^ This structure features hollow carbon spheres that offer a wealth of active sites while simultaneously preventing the agglomeration and migration of Ru/RuO_2_ nanoparticles within their internal cavities, thereby enhancing the catalyst's stability significantly (Fig. 15b and c). The experimental results indicated that the catalyst displayed outstanding OER and HER activities in an acidic medium, with its structure and composition maintaining stability after prolonged electrochemical testing. This underscores the significant role of hollow carbon sphere supports in improving the stability of Ru-based catalysts. This structural design introduces an innovative concept for advancing the development of high-performance and high-stability Ru-based electrocatalysts.

Bonding engineering emphasizes the development of robust chemical interactions between the support and the metal.^172^ Chemical bonding improves catalyst stability by preventing metal leaching *via* high bond energy interfaces. Furthermore, these interactions can modulate the electronic structure of the metal, thereby optimizing the adsorption and desorption processes of reaction intermediates and enhancing catalytic activity. This design can markedly improve catalyst performance by increasing the exposure rate of active sites and enhancing the intrinsic stability of the catalyst, particularly in complex reaction environments. Research indicates that the activity and stability of catalysts can be significantly improved by establishing strong bonds between the support and the metal.^109^ The diverse structure of Ru/MoO_2_ enhances hydrogen adsorption and desorption by facilitating the formation of Ru–O–Mo bridge bonds, which adjust the electron distribution between the metal and the support (Fig. 15d–f). The structural design facilitated the catalysts' superior HER performance across acidic, neutral, and alkaline electrolytes, while also ensuring structural and performance stability over extended usage. Experiments and theoretical calculations revealed that this bonding approach prevents the aggregation and leaching of metal particles while enhancing the adsorption capacity of the catalysts to reactants, thereby significantly improving catalytic efficiency (Fig. 15g). This approach to improving the stability and activity of catalysts *via* bonding engineering serves as a significant reference for the development of novel and efficient catalysts. A direct bonding strategy involves the introduction of sulfur-containing functional groups to serve as molecular welding points. The robust coordination capacity of sulfur functional groups facilitates the effective anchoring of the metal active center and enhances the interaction between the metal and the support, thereby improving the overall stability of the catalyst. This strategy prevents the migration and aggregation of metal particles during the reaction process, optimizes the electronic structure, and enhances catalytic performance. Yao *et al.*^173^ accomplished stable anchoring of the metal center through sulfur functional groups by synthesizing single-atom alloy catalysts (RuCo SAA/SNC) on nitrogen- and sulfur co-doped carbon nanosheets (SNC). Effective coordination between the sulfur functional groups and the metal precursors facilitated the uniform dispersion of Ru and Co atoms on the carbon supports, minimizing agglomeration. The bonding enhanced the electronic structure of the catalyst, thereby increasing its activity and stability in HER. The incorporation of sulfur functional groups optimized the local coordination environment of Ru and Co, modified the electron density, and consequently improved the migration rate of hydrogen ions and reactivity in the HER process (Fig. 15h). This bonding strategy utilizing sulfur functional groups enhanced catalyst stability and significantly improved the catalytic efficiency of the HER reaction, providing a valuable reference for the design of new high-efficiency catalysts.

Another innovative approach in bonding engineering involves the stabilization of metal oxidation states through the use of oxygen functional groups on the support surface. The coordination of oxygen functional groups effectively regulates the electronic structure of the metal center, preventing over-oxidation and thereby enhancing the stability and activity of the catalysts in acidic environments. This strategy has demonstrated significant effects in various metal-based catalysts, particularly in modulating the redox behavior of metals. Ma *et al.*^174^ achieved precise regulation of the oxidation state of Ru through the encapsulation of RuP clusters within an N, P co-doped carbon layer (NPC). This study demonstrates that robust interactions developed between the oxygen functional groups of the NPC supports and the RuP clusters. These interactions not only shielded the RuP clusters from over-oxidation in acidic conditions but also enhanced the electronic structure of Ru, resulting in superior catalytic performance in HER (Fig. 15i). The experimental results indicated that the RuP@NPC catalyst demonstrated a significantly low overpotential in a 0.5 M H_2_SO_4_ solution and maintained excellent stability during the PEM water electrolysis test for up to 200 hours, outperforming commercial Pt/C catalysts. The observed catalytic performance is primarily ascribed to the modulation of the Ru oxidation state by the oxygen functional groups of the NPC support, which optimized the Ru–H binding energy and enhanced the kinetics of the HER. This research presents compelling experimental evidence for bond engineering through the utilization of oxygen functional groups to improve catalyst performance.

## 4 Computational design and machine learning for mechanism to optimization

Theoretical simulations are essential in the advancement of carbon-supported Ru-based catalysts. Techniques such as density-functional theory, machine learning, finite-element modeling, and molecular dynamics simulations offer significant assistance in active-site engineering, reactor optimization, and stability analysis. Resolving the electronic structure of catalysts, the behavior of reaction intermediates, and the reaction mechanism at atomic and molecular levels, these computational tools inform experimental synthesis and significantly decrease trial-and-error expenses.^51,175^ Recognizing the substantial limitations that persist in contemporary modeling practices is crucial. DFT calculations typically assume ideal, defect-free surfaces and employ zero-temperature approximation, neglecting prevalent defects, solvation effects, and dynamic reconstruction observed in real catalysts. The quantity, quality, and diversity of accessible data limit machine-learning predictions. Particularly, there is a notable absence of large, standardized data sets that incorporate synthetic descriptors. Most computational studies primarily focus on intrinsic activity. The balanced portfolio necessary for industrial deployment, which includes stability, selectivity, cost-effectiveness, and scalable producibility, is usually excluded from the objective function. Current theoretical frameworks inadequately represent key industrial metrics such as synthetic complexity, catalyst lifetime, and manufacturing cost. This leads to a notable difference between the “ideal catalyst” obtained from computational models and the “practical catalyst” that can be realistically manufactured. Future intelligent design must emphasize synthesizability and stability in addition to activity, requiring the creation of genuine multi-objective optimization algorithms that successfully bridge theoretical predictions with industrial applications.

### 4.1 Role of density functional theory (DFT) in electronic structure design of catalysts

DFT serves as a crucial instrument in computational materials science, effectively linking microscopic electronic structures to macroscopic properties in the design of electrocatalysts. It is particularly crucial for the advancement of HER catalysts. Through the quantification of parameters such as charge distribution, adsorption energy, and reaction energy barriers at active sites, DFT clarifies catalytic mechanisms and provides a theoretical foundation for the design of cost-effective, high-performance catalysts. In the context of carbon-supported Ru catalysts, DFT calculations have elucidated the connection between the electronic structure of active sites, such as the position of the d-band center and charge transfer pathways, and the kinetics involved in hydrolysis and hydrogen adsorption steps. This advancement has transformed Ru-based materials from simple alternatives to Pt into distinct, high-performance catalyst systems.

**Fig. 16:**
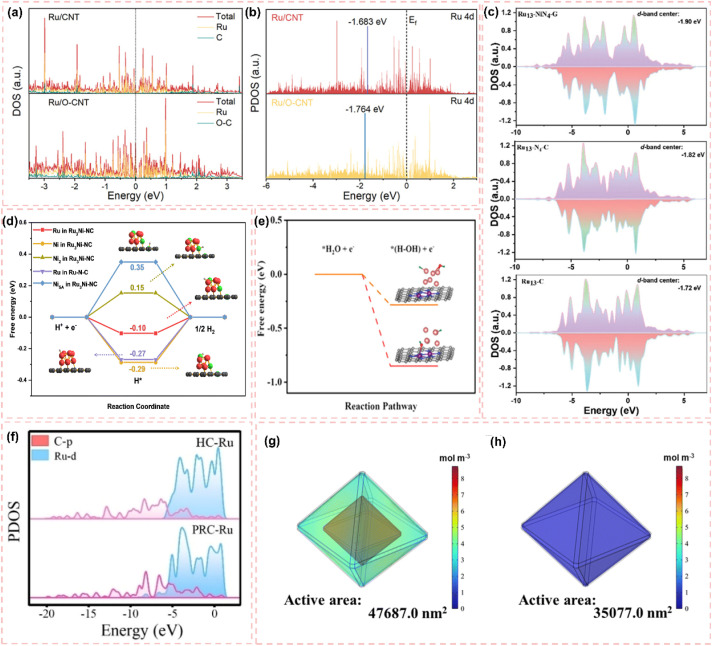
(a) The DOS plots and (b) PDOS plots of the Ru 4d orbital for Ru/CNT and Ru/O-CNT. Reproduced with permission from ref. 144. Copyright 2024, Wiley-VCH. (c) Density of states curves of the Ru/Ni–N_4_C-300 catalyst. Reproduced with permission from ref. 176. Copyright 2024, Wiley-VCH. (d) The Gibbs free energy diagram for HER at various sites. The inset shows the optimized adsorption structure of H* on various sites. Reproduced with permission from ref. 177. Copyright 2025, Royal Society of Chemistry. (e) Free energy diagram of H_2_O decomposition with OH absorption at the Ru and Co sites. Reproduced with permission from ref. 87. Copyright 2025, Royal Society of Chemistry. (f) PDOS of C, Ru for Ru@PRC and Ru@HC. Reproduced with permission from ref. 178. Copyright 2024, Wiley-VCH. Finite element method (FEM) simulation results of the H_2_ concentration in c-Ru@H-NPC (g) and c-Ru@NPC (h). Reproduced with permission from ref. 10. Copyright 2025, Royal Society of Chemistry.

DFT calculations facilitate an accurate analysis of the electronic structure of carbon-supported Ru-based catalysts. For example, determining the density of states (DOS) and energy band structures of catalysts offers insightful information about the distribution of electrons across various energy levels and their hopping behavior. Certain investigations suggest that the interplay between carbon supports and Ru nanoparticles leads to electron redistribution, resulting in a shift of the d-band center at the interface Ru sites. For example, the calculations conducted by Wang *et al.*^144^ demonstrate that oxygen-functionalized carbon nanotubes (O-CNTs) exhibit a strong interaction with Ru nanoclusters. The formation of the Ru–O–C bond facilitates electron transfer from Ru to carbon, resulting in a decrease in the d-band center of Ru (Fig. 16a and b). This reduces H* adsorption on Ru active sites, enhancing alkaline HER kinetics. The findings provide significant theoretical insights for the design of efficient alkaline HER catalysts. Zhang *et al.*^176^ developed a Ru nanocluster/single-atom Ni–N_4_ site modified nitrogen-doped carbon support (Ru/Ni–N_4_C-300) and demonstrated that the single-atom Ni sites significantly influence the electronic structure of the Ru nanoclusters. This results in a downward shift of the d-band center (Fig. 16c), which weakens the binding of H* to the Ru surface and facilitates H_2_ generation. Optimization of the electronic structure decreases the Δ*G*_H*_ of hydrolysis dissociation, thereby improving the catalytic activity for basic HER.

The researchers conducted a comprehensive analysis of the electronic structure of carbon-supported Ru-based catalysts, emphasizing the precise identification and examination of active sites. The electronic–structure properties dictate the characteristics of active sites and the pathways of catalytic reactions. Therefore, elucidating the location and mechanism of the active site is essential for comprehending catalytic reactions and improving catalyst performance. Through DFT simulations, potential active sites related to the HER were accurately identified in complex carbon-supported Ru-based catalyst structures, providing clear direction for subsequent catalyst optimization. Xu *et al.*^177^ employed DFT simulations to pinpoint potential active sites related to HER in intricate carbon-supported Ru-based catalyst structures, providing valuable insights for subsequent optimization efforts. The simulations demonstrated that Ni single atoms influence the interfacial water structure, leading to a decrease in the energy barrier for water dissociation and an increase in the rate of the Volmer step. The robust interaction between alloyed Ni and Ru enhances the electronic structure of the Ru site, aligning the Δ*G*_H*_ more closely with the thermoneutral value and promoting the kinetics of the Heyrovsky step (Fig. 16d). This study clarifies the synergistic mechanism between Ni single atoms and RuNi alloy nanoclusters, offering a theoretical basis for the design of efficient alkaline HER catalysts. It also emphasizes the advantages of single-atom engineering and alloying strategies in the context of industrial green hydrogen production. This DFT-based method for identifying active sites provides valuable theoretical insights for the design and optimization of complex catalyst systems.

Following the identification of the active site, it is crucial to examine the catalytic reaction mechanism to enhance comprehension of the catalyst's functionality. DFT calculations provide a reliable method for clarifying the HER mechanism on carbon-supported Ru-based catalysts. The entire HER process can be meticulously simulated, encompassing H_2_ adsorption, dissociation, H atom recombination, and desorption. DFT offers accurate modeling and energy calculations for reaction intermediates and transition states, yielding important insights into the catalytic mechanism. DFT calculations have revealed the distinct mechanism of single-atom CoN_4_-supported Ru-based catalysts in alkaline HER (Fig. 16e).^87^ The catalyst's effectiveness arises from the dual modulating function of the CoN_4_ support: it alleviates OH_ad_ blockage on Ru active sites through competitive adsorption and reduces the OH delocalization energy barrier on Ru's surface *via* electronic regulation. This dual action optimizes the catalytic pathway, improving both activity and stability. DFT simulations of electron transfer, adsorption behavior, and energy variations support the observed high performance in experiments. The carbon support stabilizes Ru nanoclusters and CoN_4_ sites, while its strong interaction with Ru and Co optimizes the electronic structure. The result enhances the electrical conductivity and charge transfer of the catalyst, thereby increasing catalytic activity.

With an increasing comprehension of catalytic mechanisms, researchers are able to forecast and improve catalyst performance informed by this insight. DFT calculations serve as effective instruments for forecasting and enhancing the performance of carbon-supported Ru-based catalysts. Through the manipulation of catalyst composition, the selection of carbon support type and structure, as well as the adjustment of Ru nanoparticle size and morphology, it is possible to develop theoretical models that evaluate the efficacy and durability of the catalysts across various conditions. Gong *et al.*^178^ examined the interaction between pentagonal carbon ring (PRC)-rich carbon materials and Ru nanoparticles through DFT calculations, influencing HER activity. Pentagonal carbon-ring defects were found to modulate the electronic structure of carbon supports. This process decreases the band gap, optimizes the p-band center, and enhances hydrogen adsorption free energy, thereby improving overall HER performance (Fig. 16f). The anchoring of Ru nanoparticles on PRC-rich carbons results in p–d orbital hybridization between C and Ru atoms, leading to electron redistribution and the formation of electron-deficient Ru nanoparticles. This step reduces the binding of hydrogen intermediates and enhances HER activity. Theoretical predictions provide robust support for the design of new high-efficiency catalysts. Experimental validation demonstrates that the PRC-Ru synergy displays exceptional HER activity and stability across various pH levels, facilitating the development of cost-effective, high-efficiency catalysts for green hydrogen production.

The metal–support interaction (MSI) is essential in the prediction and optimization of catalyst performance based on DFT. This interaction affects the electronic structure of the catalyst and has a substantial effect on the formation of active sites and the mechanisms of reaction. An in-depth examination of MSI can reveal mechanisms for enhancing catalyst performance and provide robust theoretical support for the precise regulation of catalyst efficacy. Research on lignin-derived carbon-supported N/S-*co*-doped Ru-based electrocatalysts indicates that sulfur defects markedly enhance metal–carbon electronic interactions, thereby improving both catalyst activity and stability.^179^ This interaction modifies the electronic structure of the catalyst and optimizes hydrogen adsorption energy, leading to superior HER performance in both acidic and basic environments. In Ru-doped nickel nanoparticle catalysts supported on carbon nanofibers with graphitic carbon shells, robust metal–support interactions inhibit thermal aggregation, thereby mitigating specific surface area loss and facilitating charge transfer.^126^ DFT calculations indicate that these interactions improve catalyst activity by altering the metal's electronic structure, thereby enhancing the HER reaction synergistically. The studies emphasize the significance of integrating metal–support interactions with DFT calculations in the design of electrocatalysts, presenting novel strategies for the development of highly efficient electrocatalysts.

In summary, DFT calculations are indispensable in researching carbon-supported Ru-based HER catalysts. This methodology offers a robust resource for acquiring a comprehensive insight into the connection between catalyst structure and performance. The result contributes to the design and development of carbon-supported Ru-based catalysts that exhibit enhanced activity, stability, and cost-effectiveness, thus propelling the progress of efficient and sustainable energy conversion technologies.

### 4.2 Application of finite element simulation in electrode structure optimization

The performance of a catalyst in the HER system is influenced by both the intrinsic activity of its active sites and the mesoscopic structure of the electrode, which encompasses the pore network, mass transfer pathways, and conductive channels. The finite element simulation (FEM) integrates electrochemical, hydrodynamic, and thermodynamic equations, providing a theoretical framework to tackle mass transfer limitations at elevated current densities.^180^ The analysis focuses on quantifying spatial variations in current distribution, concentration gradients, and reaction rates within the electrode. In carbon-supported Ru-based catalyst systems, FEM plays a vital role in enhancing the 3D electrode structure and fully utilizing the potential of Ru active sites. This contributes to the realization of extensive industrial hydrogen production.

The investigation of carbon-supported Ru-based catalysts reveals that FEM provides a solid theoretical foundation for comprehending the relationship between mesostructure and catalyst performance. Simulations reveal that mesostructural elements, including pore networks, mass-transfer pathways, and conductive channels, significantly influence the overall catalyst performance. FEM simulations showcase the advantages of hollow catalysts in enhancing hydrogen-diffusion efficiency compared to solid structures. They elucidate the mass-transfer mechanism by demonstrating that pores in hollow structures offer supplementary pathways for hydrogen molecules, hence diminishing hydrogen buildup and concentration polarization while enhancing reactant renewal (Fig. 16g and h).^10^ The simulation results closely correspond with experimental observations, confirming the efficacy of FEM in catalyst design.

**Fig. 17:**
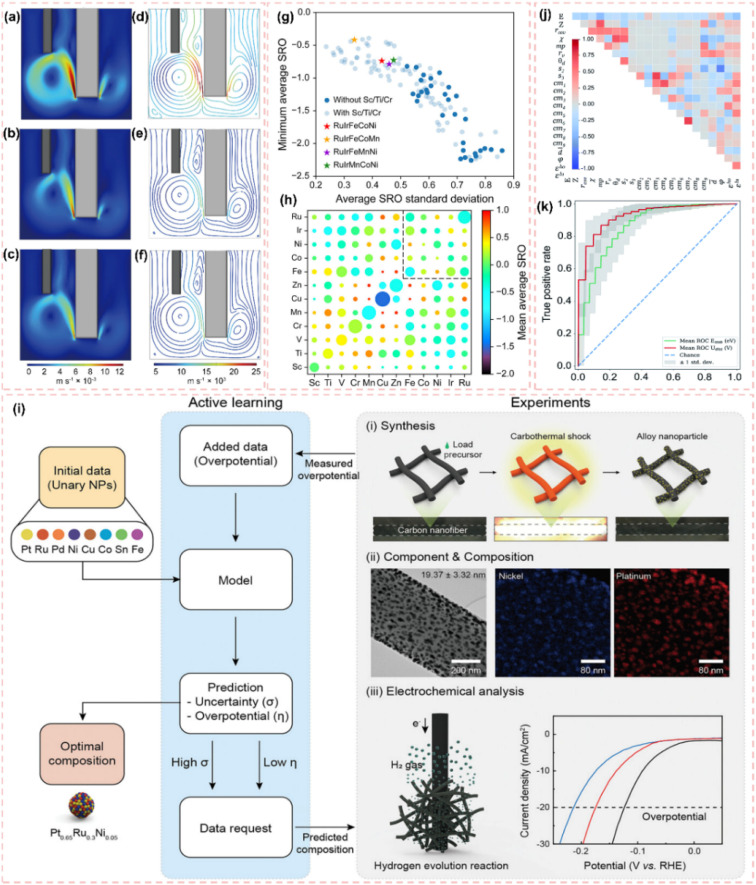
Simulation result of the hydrogen production rate for (a) Ru-NBC, (b) Ru-NC-1, and (c) Ru-NC-2. Surface tension of hydrogen bubbles on the cathodic (d) Ru-NBC, (e) Ru-NC-1, and (f) Ru-NC-2. Reproduced with permission from ref. 111. Copyright 2025, American Chemical Society. (g) Scatter plot of the minimum average SRO *vs.* the standard deviation of average SRO and (h) correlation plot of the mean (indicated by circle color) and standard deviation (indicated by circle radius) of the average SRO (from the last 3000 REMD/MC steps), for atom pairs in equimolar RuIr-based bulk HEAs with three other elements chosen from all 3d metals. Reproduced with permission from ref. 185. Copyright 2025, American Chemical Society. (i) The overall workflow of searching for low-overpotential multi-metallic alloy catalysts. Reproduced with permission from ref. 186. Copyright 2022, Wiley-VCH. (j) The heatmap of Pearson correlation coefficient matrix among the selected 21 features/descriptor. The intensity of colors represents the direct correlation (E: H-adsorption/free energy). (k) Receiver operating characteristic (ROC) curve for ERT-classifier and the corresponding area under the curve (AUC) for classification of *E*_stab_ and *U*_diss_. The mean ROC is calculated among curves for 100 random splits into the training and validation sets for predicting the thermodynamic and electrochemical stabilities. Reproduced with permission from ref. 187. Copyright 2022, Royal Society of Chemistry.

FEM has been extensively utilized in catalyst research. It enables researchers to thoroughly investigate catalyst reaction processes and structure–performance correlations. Simulations enable researchers to gain a thorough understanding of variations in catalyst performance across diverse structures and conditions. This offers comprehensive theoretical and technical direction for the design and optimization of efficient catalysts. Feng *et al.*^111^ employed finite element simulation to investigate catalyst reaction processes, demonstrating the influence of hydrogen-bubble behavior on fluid flow. Their findings indicated that the formation and detachment of hydrogen bubbles enhance local airflow velocity, resulting in velocity differentials with the surrounding electrolyte and the generation of vortices and eddies (Fig. 17a–f). This facilitates mass transfer and enhances HER performance. The research focused on the importance of catalyst-surface hydrophilicity in enhancing HER efficiency, indicating that increased hydrophilicity results in enhanced hydrodynamic forces and accelerated HER kinetics. The findings are consistent with earlier finite element simulation studies, highlighting the technique's essential function in connecting catalyst meso-structure to macro-performance.

Exploring various substrate materials is essential in HER catalyst research for understanding the diversity of catalyst properties. Various substrates, including carbon and metal, possess distinct physicochemical properties that markedly affect catalyst activity, stability, and mass-transfer efficiency. Carbon supports are extensively researched due to their superior electrical conductivity, high specific surface area, and chemical stability while other substrate materials also deserve consideration. For example, an investigation into electrodes featuring nanotip structures, while not carbon-based, employed finite element simulation to uncover the distribution of electric fields and ion concentrations.^181^ This insight acts as a valuable reference for the analysis of Ru-based catalysts. The nanotip structure generates a focused electric field, facilitating the Marangoni effect and improving bubble detachment and HER kinetics. This method of regulating the interfacial microenvironment presents an innovative strategy for the design of efficient HER electrodes that are universally applicable across different pH levels. While the substrate in this investigation varies from carbon, the finite element simulation approach and findings provide valuable insights for comprehending and enhancing the efficacy of Ru-based catalysts.

FEM is essential in the analysis of HER catalysts, serving as a significant tool for elucidating the connection between catalyst structure and performance. The application of Ru-based carbon catalysts remains in the preliminary exploration stage. However, existing research indicates significant potential. FEM offers substantial theoretical support for catalyst design and performance enhancement by optimizing the microstructure of catalysts, analyzing the impact of bubble behavior on fluid flow, and elucidating the influence of various substrate materials on the distribution of electric fields and ion concentrations. Nonetheless, the present investigation has merely revealed the surface of the potential for finite element simulation within this domain. To enhance the comprehension and utilization of Ru carbon catalysts, it is essential to pursue innovative studies and explore a variety of research concepts moving forward. Through the integration of experimental data and simulation analysis, a more thorough understanding of catalyst performance optimization can be achieved by conducting a detailed exploration of the mechanisms involved in multi-physical field coupling, the relationship between complex microstructures and macroscopic performance, and the behavior of catalysts across various operating conditions. This will not only advance the practical use of Ru carbon catalysts in energy sectors like hydrogen production from electrolyzed water but also offer significant theoretical resources and innovative concepts for the progression of the entire electrocatalysis domain.

### 4.3 Utilizing machine learning in catalysis forecasting and development

The conventional approach to catalyst design largely relies on trial-and-error techniques and empirical insights, both of which are time-intensive and laborious. This method poses challenges in swiftly and precisely pinpointing high-performance catalysts in intricate, evolving material systems. Nonetheless, the swift progress in big data, high-performance computing, and artificial intelligence has opened up fresh avenues for the design and optimization of carbon-supported Ru-based catalysts through machine learning. Machine learning (ML) has the capability to reveal patterns in data, develop effective predictive models, and enhance the processes of catalyst screening and design.^182^ This offers substantial backing for improving the efficacy of carbon-supported Ru-based catalysts and signals a transformative shift in the field of catalyst innovation and advancement.

The field of catalyst design is presently experiencing a significant transformation propelled by ML technology.^183^ This technology functions as an accurate “translator” that connects the microscopic electronic structure with macroscopic catalytic performance. The crucial advancement is found in creating a quantitative link between the two, with “descriptors” serving as the essential components of this relationship.^184^ Through a thorough exploration and strategic application of descriptors, ML models can perform a comprehensive analysis of the physicochemical mechanisms of alloy catalysts, grounded in their distinct electronic structural characteristics. This facilitates precise predictions of macroscopic catalytic performance, which is vital to the systematic design of multi-component alloy catalysts. This leads to a notable reduction in the research and development cycle, minimizes costs associated with trial and error, and advances the entire field towards enhanced intelligence and efficiency, enabling the attainment of high-precision predictions regarding macroscopic catalytic performance.

ML has become an effective instrument in the design of catalysts, especially for those based on Ru. The distinctive electronic structure of Ru provides remarkable catalytic activity. However, peroxidative solvation associated with lattice oxygen participation hinders its stability. Conventional research and development approaches face challenges in optimizing Ru catalysts both efficiently and cost-effectively. Nonetheless, machine learning has significantly altered this domain. In Ru_*x*_(Ir, Fe, Co, Ni)_1−*x*_ multi-component alloy catalysts, ML-informed force fields (MLIP) and molecular dynamics simulations have revealed atomic-scale mixing behaviors, surface-biased aggregation, and phase-formation rules.^185^ Experimental and theoretical findings demonstrate that near-equimolar alloys develop multiphase structures, primarily consisting of face-centered cubic (fcc), and to a lesser extent, hexagonal close-packed (hcp) phases (Fig. 17g and h). This unique microstructure provides remarkable activity and stability to the catalyst. That significant advancement underscores the ability of machine learning to improve the performance of Ru catalysts through the precise adjustment of their electronic and crystal structures, tackling stability challenges, and providing a framework for the development of efficient, cost-effective Ru-based catalysts. This approach is expected to transform Ru-catalyst development and enable their extensive use in industrial hydrogen production through water electrolysis.

**Fig. 18:**
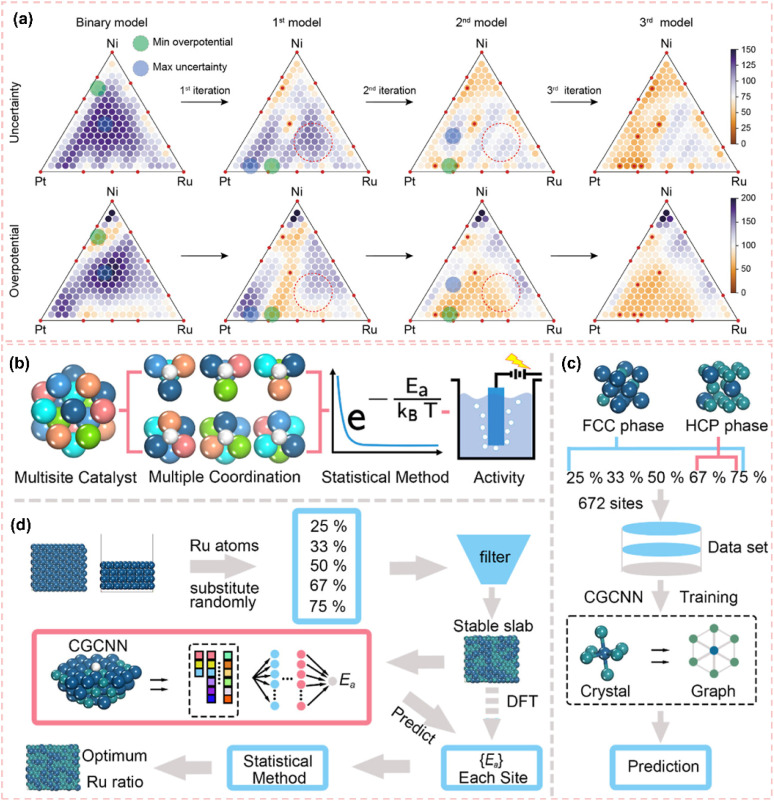
(a) Active-learning results for a ternary composition. With each iteration, a triangular diagram of uncertainty and overpotential is updated for the Pt–Ru–Ni system. The red dotted circles indicate the change in prediction without data added at certain points after iteration. Reproduced with permission from ref. 186. Copyright 2022, Wiley-VCH. Schematic of the conceptual framework and calculation flow. (b) Illustration of multisite alloy catalyst model. (c) Training process of CGCNN model. (d) Overall calculation flow. Reproduced with permission from ref. 188. Copyright 2024, American Chemical Society.

With the increasing adoption of ML techniques in catalyst development, an expanding array of studies is investigating their potential across various catalytic systems. Kim *et al.*^186^ introduced a novel screening strategy for HER catalysts that integrates active learning with experimental data. The machine learning model iteratively updates itself, reduces the optimization space, and enhances screening efficiency while minimizing costs (Fig. 17i). This protocol can be widely applied to other families of electrocatalysts by adjusting both the identity and the ratio of constituent elements. Fig. 18a illustrates that training the model exclusively on binary data results in significant uncertainty. However, updating the model across an extensive compositional range substantially decreases the uncertainty for Pt–Ru–Ni samples and achieves the lowest overpotential. The model predicts the HER overpotentials of multi-metallic alloys solely based on precursor-metal ratios, eliminating the need for quantum-chemical calculations and identifying optimal compositions that include Ru. The Pt_0.65_Ru_0.3_Ni_0.05_ ternary demonstrates an overpotential of only 54.2 mV, surpassing that of pure Pt and validating the potential of machine learning in enhancing Ru-catalyst development. Umer *et al.*^187^ performed a systematic investigation of different transition metal single-atom catalysts utilizing DFT in conjunction with ML (Fig. 17j and k). The findings revealed that Ru@N_2_C_2_ catalysts, created by embedding Ru single atoms within N_2_C_2_ substrates, exhibited exceptional HER activity and stability. Specifically, Ru@N_2_C_2_ demonstrated a nearly thermoneutral Δ*G*_H*_ for hydrogen adsorption (Δ*G*_H*_ ≈ 0.02 eV), suggesting ultralow overpotential and effective hydrogen generation. The catalyst demonstrated remarkable structural stability, with thermodynamic stability energy and electrochemical dissolution potential aligning with stability requirements. This indicates that Ru@N_2_C_2_ is capable of sustaining its catalytic efficiency over time, free from problems such as metal aggregation or dissolution. The results underscore Ru@N_2_C_2_ as an exceptionally promising electrocatalyst for HER.

The application of ML was pivotal in analyzing and forecasting catalyst performance based on vast experimental datasets, informing experimental design and highlighting its essential role in the advancement of catalysts. Zhou *et al.*^188^ employed the CGCNN model to accurately predict the hydrogen adsorption free energy across various sites on Pt–Ru alloy surfaces and proposed the equivalent site ratio as a quantitative measure of overall activity. The study indicates that HER performance is optimal with Ru content between 20% and 30%, consistent with experimental trends, thereby underscoring the accuracy of ML models in correlating microscopic sites with macroscopic catalytic performance (Fig. 18b–d). In the study by Jiang *et al.*,^189^ developed a quantitative predictive model that effectively identified a combination of C–N, C–C, Fe–N, Ru–O, and C–P bonds as a promising candidate for bifunctional activity due to its high frequency of occurrence and the lowest predicted Δ*E* value. The experimental results provided robust support for the theoretical prediction, as the synthesized catalysts exhibited notable bifunctional activity. The results highlight the distinct benefits of AI in catalyst design and offer considerable motivation for the advancement of bifunctional catalysts. Furthermore, they create a novel pathway for the innovation of Ru-based catalysts and are anticipated to enhance Ru's application potential in catalysis.

ML has shown considerable promise in the advancement of carbon-supported Ru-based catalysts for HER studies. This approach transforms conventional methods in catalyst exploration and innovation by facilitating data-informed accuracy in material evaluation, structural enhancement, performance assessment, and the discovery of novel materials, effectively transcending empirical limitations. With the refinement of ML algorithms, the precision and breadth of data acquisition, along with the expansion of computational resources, this technology is set to significantly influence the design and optimization of carbon-supported Ru catalysts. It will propel ongoing advancements in HER technology and the wider energy transition sector.

ML exhibits considerable potential in screening and predicting catalysts. However, most existing research remains trapped in the “black-box” prediction phase. The absence of physical interpretability hinders researchers from deriving universal physical or chemical principles from machine learning predictions, which could inform experimental design. The development of understandable AI and the deep integration of ML with first-principles analysis are essential. This approach should elucidate not only the effective elements but also the underlying reasons, which will be essential for the advancement of AI-driven catalyst design.

## 5 Conclusions and outlook

**Fig. 19:**
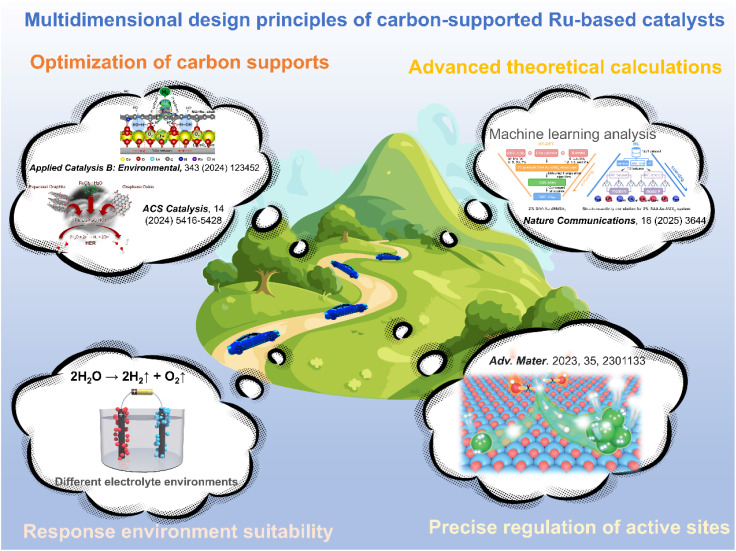
Summary of design principles for carbon-supported Ru-based catalysts.

Carbon-supported Ru-based catalysts show significant potential for use in the HER. Clarifying their structure–activity relationships and setting up logical design principles is essential for creating effective catalysts. This review systematically analyzes the complex design of carbon-supported Ru-based HER catalysts, establishing a comprehensive framework that integrates environmental adaptability, active site engineering, support optimization, and advanced computational design (Fig. 19). The transition from bulk Ru to precisely engineered single atoms, clusters, and heterostructures underscores the movement towards atomic-level precision in catalyst design. Carbon supports have transitioned from passive scaffolds to active components, with their structure, dimensions, and surface chemistry significantly affecting Ru's electronic structure, mass transfer efficiency, and overall catalytic stability. The integration of advanced theoretical modeling and machine learning is transforming discovery and optimization processes, overcoming the limitations of traditional trial-and-error methods. Despite significant progress, the industrial application of carbon-supported Ru-based catalysts still faces challenges. Critical analyses identify significant discrepancies between academic research and practical needs that future investigations should address.

(1) The validity of stability assessments is challenged, as the majority of literature derives stability conclusions from mild and idealized laboratory tests characterized by low current density, pure electrolytes, stable temperatures, and short-term operations. This stands in stark contrast to the challenging conditions faced by industrial water electrolyzers, which function at elevated current densities (≥1 A cm^−2^), experience frequent start-stop cycles, endure temperature variations, and are subjected to impurity ions. Under these rigorous conditions, Ru species are susceptible to dissolution, migration, aggregation, and oxidative passivation. Carbon supports may experience electrochemical corrosion or mechanical degradation, leading to a significant decline in performance. Future research should focus on assessing catalyst performance in conditions that are relevant to industrial applications. Comprehending degradation mechanisms in intricate environments is essential for the development of next-generation catalysts that exhibit genuine long-term durability.

(2) The bottleneck is in scalability and cost synthesis. The field contains numerous high-performance catalysts developed through complex, multi-step processes that include precise wet chemistry, controlled atmosphere deposition, or intricate template removal. Although effective in laboratory settings, these methods frequently exhibit low yield, variability between batches, elevated costs, and considerable difficulties in scaling up. The shift from gram-scale synthesis in research laboratories to kilogram-scale production for industrial application constitutes a significant challenge. Future research should focus on the fundamental kinetics of nucleation and growth of Ru species. This comprehension will inform the creation of robust, reproducible, and scalable synthesis strategies, including continuous-flow reactors, advanced impregnation techniques, and gas-phase synthesis methods. Investigating cost-effective and stable Ru precursors, along with developing efficient recycling protocols for spent catalysts, is essential for improving economic viability and sustainability.

(3) The current understanding of dynamic interfaces and reaction mechanisms remains superficial. The HER is a dynamic process characterized by the continual alteration of the catalyst's surface structure, oxidation state, and the interfacial water network, all of which are affected by the applied potential. Current *in situ* characterization techniques, although effective, frequently exhibit limitations in spatiotemporal resolution and information depth, hindering a comprehensive understanding of the active site during operation, the dynamic adsorption/desorption behavior of H*, and the structure of the interfacial solvation layer. Current research frequently exhibits inadequate mechanistic validation for intricate processes. Claims regarding hydrogen spillover effects often lack rigorous, multi-criteria validation and remain speculative. Future research should combine *in situ* characterization with theoretical calculations to yield direct and conclusive evidence for complex multi-step reaction mechanisms. This will provide a more robust scientific basis for catalyst design.

(4) The process involves integrating theoretical frameworks and experimental methodologies, while also addressing the challenges posed by data scarcity. Computational tools such as DFT and FEM offer significant insights. However, they frequently depend on simplified, idealized models that do not adequately represent the heterogeneity and complexity of actual catalysts. The integration of theoretical predictions with experimental validation is inadequate, restricting the ability of theory to offer precise and actionable guidance for catalyst optimization. An absence of large, standardized, and high-quality datasets hinders the field, significantly limiting the advancement of robust and predictive machine learning models. Future research should prioritize the development of a comprehensive, open-access database that encompasses catalyst syntheses, properties, and performance metrics. The integration of multi-scale modeling, which includes solvation and electric field effects, with understandable machine learning based on high-fidelity data is essential for enhancing the rational design of catalysts and forecasting their long-term performance evolution.

In conclusion, carbon-supported Ru-based catalysts exhibit significant potential as alternatives to Pt in the hydrogen evolution reaction. Overcoming existing limitations necessitates a collaborative, multidisciplinary approach. The emphasis should transition from exclusively seeking increased activity in idealized environments to the development of catalysts that demonstrate efficiency, stability, scalability, and economic viability. Through the adoption of stringent testing protocols, advancements in scalable synthesis, exploration of the dynamic interface, and enhanced integration of data science with both experimental and theoretical chemistry, the field can address these challenges effectively. This coordinated initiative will be crucial in maximizing the potential of Ru-based catalysts, significantly advancing the development of a green hydrogen economy.

## Author contributions

All of the authors contributed to the manuscript preparation. Z. L. and T. Y. conceived the outline of the manuscript. Z. L., S. Z., B. L., and P. W. wrote the original draft of the manuscript. Z. L., S. Z., and T. Y. discussed and helped revise the manuscript.

## Conflicts of interest

There are no conflicts to declare.

## Data Availability

No primary research results, software or code have been included and no new data were generated or analysed as part of this review.
